# Multifunctional metal-organic framework (MOF)-based nanoplatforms for cancer therapy: from single to combination therapy

**DOI:** 10.7150/thno.80687

**Published:** 2023-01-01

**Authors:** Jie Yang, Dihua Dai, Xi Zhang, Lesheng Teng, Lianjun Ma, Ying-Wei Yang

**Affiliations:** 1 School of Life Sciences and College of Chemistry, Jilin University, 2699 Qianjin Street, Changchun 130012, P. R. China.; 2 Department of Endoscopics, China-Japan Union Hospital of Jilin University, Jilin University, Changchun 130012, P. R. China.

**Keywords:** biomaterials, cancer therapy, combination therapy, metal-organic frameworks, porous materials

## Abstract

Cancer remains a severe threat to human health. To date, although various therapeutic methods, including radiotherapy (RT), chemotherapy, chemodynamic therapy (CDT), phototherapy, starvation therapy, and immunotherapy, have entered a new stage of rapid progress in cancer theranostics, their limited therapeutic effect and significant side effects need to be considered carefully. With the rapid development of nanotechnology, the marriage of nanomaterials and therapeutic methods provides the practical possibility to improve the deficiencies in cancer therapy. Notably, metal-organic frameworks (MOFs) composed of ions/clusters and bridging ligands through coordination bonds have been widely applied in cancer therapy to deal with the drawbacks of different therapeutic methods, such as severe side effects, low stability, and poor efficacy, owing to their controllable morphologies, tailorable diameters, diverse compositions, tunable porosities, high specific surface areas, facile functionalization, and good biocompatibility. This review summarizes the recent advanced developments and achievements of multifunctional MOF-based nanoplatforms for cancer therapy through single therapy methods, including RT, chemotherapy, CDT, phototherapy (photodynamic and photothermal therapy), starvation therapy and immunotherapy, and combination therapy methods. Moreover, the prospects and challenges of MOF-based nanoplatforms used in tumor therapy are also discussed.

## 1. Introduction

Cancer, a type of intractable disease with a high recurrence rate, high fatality rate, and easy metastasis, is one of the greatest threats to human health that causes millions of deaths every year 1. Currently, surgery, radiotherapy (RT), and chemotherapy are still the universal strategies for tumor therapy, but their disadvantages of poor precision, low efficiency, and grievous side effects seriously weaken the therapeutic effect of tumors 2-4. To date, numerous therapeutic methods, such as chemodynamic therapy (CDT) 5,6, phototherapy 7,8, starvation therapy 9, and immunotherapy 10,11, possessing favorable features of low side effects, non-invasiveness, and facile operation, have been widely developed to improve the anticancer effect for meeting the growing demands of clinical treatment. However, these treatment strategies still face severe challenges during cancer therapy. For example, CDT suffers from the limitation of H_2_O_2_ content in the tumor microenvironment (TME). Photodynamic therapy (PDT) shows poor stability of photosensitizers and high dependence on O_2_. Therefore, researchers are committed to developing suitable approaches to improve the therapeutic effects during tumor management. In the recent few decades, the continuous development of nanomaterials has brought new ideas to ameliorate the drawbacks of various tumor therapy methods. Consequently, different advanced materials with superior properties have been employed in tumor therapy to access the merits of high efficiency, outstanding precision, and negligible side effects 12-16.

Metal-organic frameworks (MOFs) - an exciting and valuable class of porous organic-inorganic coordinated materials constructed from metal ions/ion clusters as nodes and organic molecules as ligands - have been widely fabricated and applied for cargo/drug delivery and cancer therapy due to their unique superiorities, including controllable morphologies, tailorable diameters, diverse compositions, tunable porosities, high specific surface areas, facile functionalization, and favorable physicochemical properties 17-22. Since Férey and coworkers first reported Materials of Institut Lavoisier (MIL)-100 and MIL-101 for the delivery of the drug ibuprofen in 2006 23, various MOF-based nanoplatforms have been designed and constructed in recent decades to meet the diverse needs of cancer therapy. For example, MOF-based nanoplatforms constructed from or loaded with high Z element such as Hf^4+^ are widely applied in tumor radiotherapy. MOF-based nanoplatforms bearing catalytic activity exhibit unique advantages in both CDT and PDT. In addition, MOF-based nanoplatforms composed of porphyrin or porphyrin derivatives provide an excellent option for cancer therapy through PDT. Notably, MOFs exhibit the following distinct advantages in tumor therapy: ⅰ) the combination of thousands of metal ions/ion clusters and bridged ligands provides customizable options to construct multifunctional MOF-based nanoplatforms for cancer therapy; ⅱ) high porosities and large specific surface areas can guarantee the efficient loading of functional agents such as chemotherapeutic drugs, ions, phototherapeutic agents, proteins, enzymes, and antigens 24,25; ⅲ) appropriate diameters endow MOF-based nanoplatforms with passive targeting to tumor sites through a size-dependent enhanced permeability and retention (EPR) effect; ⅳ) through the coordination effect of the metal nodes and the covalent coupling by organic ligands bearing functional groups (such as -COOH, -NH_2_, -N_3_) on the surface of MOFs, some unique entities like active targeting molecules, supramolecular nanovalves, biomacromolecules, polymers, and cell membranes, are introduced onto the surface of MOFs to improve the targeting effect, colloidal/circulation stability, biocompatibility, and stimuli responsiveness during cancer therapy 26-30; ⅴ) the degradability triggered by the TME facilitates the efficient excretion of MOFs, which indicates the great potential of MOF-based nanoplatforms for cancer therapy using different therapeutic methods.

**Scheme 1:**
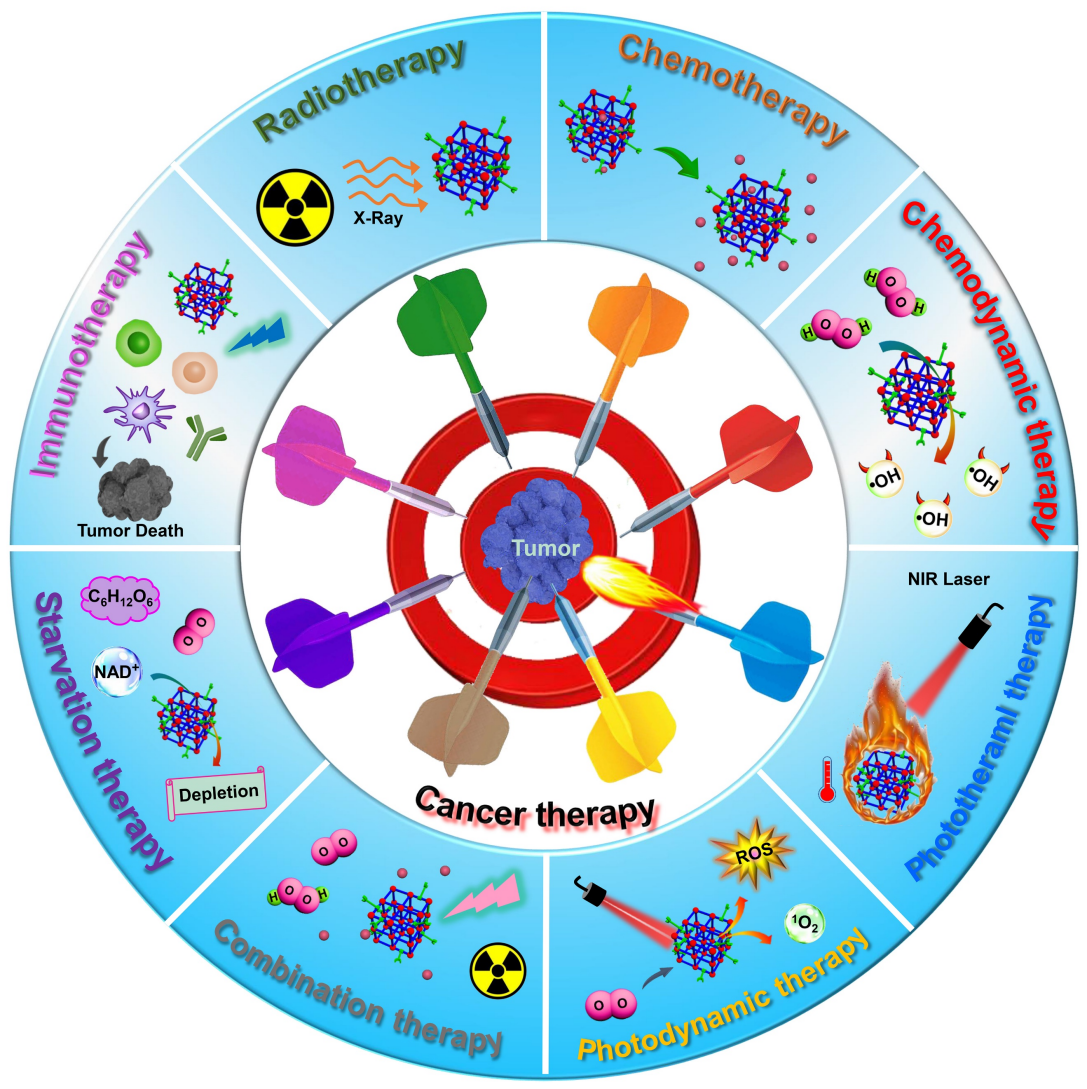
Schematic diagram of MOF-based nanoplatforms for efficient cancer theranostics through single therapy, including RT, chemotherapy, CDT, PTT, PDT, starvation therapy and immunotherapy, and combination therapy.

In this review, we mainly focus on the recent advanced developments and achievements of representative MOF-based nanoplatforms for efficient cancer treatment employing single therapy methods, including RT, chemotherapy, CDT, phototherapy (PTT and PDT), starvation therapy and immunotherapy, and combination therapy methods (**Scheme 1**). The effectiveness and necessity of MOF-based nanoplatforms in tumor therapy are highlighted through the interpretation of the representative studies. Furthermore, the prospects and challenges of MOF-based nanoplatforms used in various tumor therapy methods are also envisaged. In contrast to previous reviews that discuss the applications of MOFs in cancer therapy, this review will highlight the customizable construction strategies and therapeutic mechanisms of MOF-based nanoplatforms for cancer therapy from single therapy to combination therapy and provide an outlook for the potential application of MOF-based nanoplatforms in the clinical treatment of cancer, hoping to further promote the development of MOF-mediated cancer therapy.

## 2. Single therapy based on MOFs

Practical methods, including RT, chemotherapy, CDT, phototherapy, starvation therapy, and immunotherapy, provide alternative strategies for diverse cancer therapy 31,32. However, their limited therapeutic efficacy, severe side effects, and low targeting seriously reduce the therapeutic efficacy of tumors. Over the past few decades, researchers have constructed numerous nanomaterials to tackle the problems of the abovementioned cancer treatment methods to increase therapeutic efficacy 33-35. Unfortunately, although certain success has been achieved in cancer therapy, some emerging issues must be considered carefully. Compared with traditional pure organic or inorganic nanomaterials, MOFs have become one of the most promising candidates for efficient cancer therapy due to their intrinsic features of large specific surface area, tunable pore sizes, customizable diameters, diverse composition, easy post-synthetic modification, and good degradability in the TME. MOFs can deliver various therapeutic agents, such as drugs, enzymes, proteins, photosensitizers, photothermal agents, and antibodies, to target sites, improving tumor treatment efficiency and reducing side effects 36. Besides, the strategy of introducing functional nanocomponents such as Au nanoparticles (Au NPs), Fe_3_O_4_ NPs, CuS NPs, and polypyrrole NPs in MOFs to develop intelligent and multifunctional nanoplatforms has been applied to achieve precise cancer therapy 37-40. Abundant investigations have verified that MOF-based nanoplatforms can serve as desired candidates for efficient cancer therapy. In this section, we will outline the applications of MOF-based nanocomposites as smart nanoplatforms for tumor therapy through single therapy strategies of RT, chemotherapy, CDT, phototherapy, starvation therapy, and immunotherapy (**Table 1**). Meanwhile, the drawbacks and superiorities of these therapeutic methods are also summarized (**Table 2**).

### 2.1 Radiotherapy

RT, as a classic and effective tumor treatment method with the superiorities of deep penetration and good ability to eliminate tumor cells, uses high doses of ionizing rays to irradiate the tumor sites, achieving local cancer cells elimination and tumor cell growth inhibition 43,71. However, RT exhibits poor therapeutic effects on tumors in clinical applications, which is caused by the lack of specific killing ability of tumor cells. Meanwhile, due to the low absorption rate of radiation by tumor tissue, high radiation doses are usually required to effectively inhibit tumor growth, which increases the risk of systemic toxic side effects. Long-term RT may cause the thickening of blood vessels and some diseases, such as atherosclerosis and thrombosis 72. In addition, multiple RT will induce the resistance effect of tumor cells to radiation, reducing the effect of RT-mediated tumor therapy. Generally, MOF-based nanoplatforms constructed from high Z elements or functionalized with high Z element nanoparticles possess strong X-ray attenuation abilities and high X-ray absorption coefficient, which can emit secondary electrons (such as Compton electrons, photoelectrons, and Auger electrons) upon X-ray irradiation, resulting in the ionization of intracellular components and water to achieve radiation sensitization. Meanwhile, they can interact with ionizing radiation and convert intracellular O_2_ into reactive oxygen species (ROS), increasing tumor sensitivity and inducing tumor cell apoptosis. Thus, to improve the efficacy of radiotherapy, MOF-based nanoplatforms as outstanding candidates have been widely used in RT of tumors to enhance the therapeutic effect and reduce the systemic side effects through the following approaches 73. ⅰ) MOFs composed of metal ions/clusters containing high Z elements (such as Hf-MOF) are used as radiosensitizers to improve RT efficacy due to the strong X-ray attenuation ability of high-Z metal atoms; ⅱ) some inorganic NPs with high Z elements can be doped in MOFs to construct MOF-based nanocomposites, thus enhancing tumor treatment effect through the radiosensitization; ⅲ) due to the high porosity, suitable pore sizes, and good biocompatibility, MOFs are considered as ideal nanocarriers for the loading of radiosensitizer molecules to improve RT efficiency.

**Figure 1:**
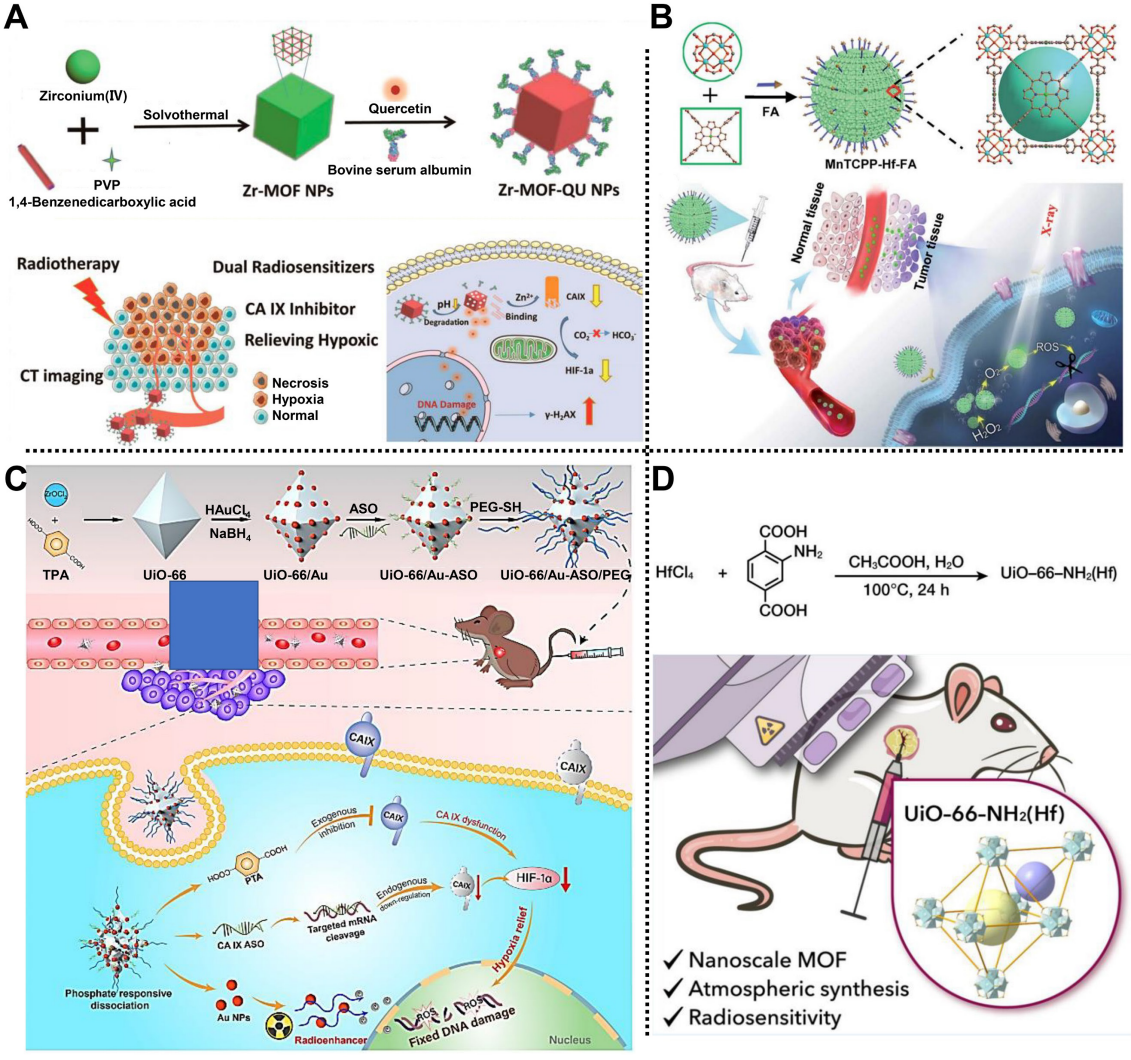
** (A)** Schematic representation of the fabrication of Zr-MOF-QU and its application in RT. Adapted with permission from 41. Copyright 2019, American Chemical Society. **(B)** Schematic description of MnTCPP-Hf-FA applied to enhance hypoxia-induced radioresistance. Adapted with permission from 42. Copyright 2019, Royal Society of Chemistry. **(C)** The preparation process of UAAP and its application in cancer therapy by RT. Adapted with permission from 38. Copyright 2021, Elsevier. **(D)** Schematic description of UiO-66-NH_2_(Hf) applied to enhance the RT effect in cancer therapy. Adapted with permission from 43. Copyright 2022, American Chemical Society.

In 2019, Meng, Tan, Chen, and coworkers prepared a multifunctional nanocomposite based on Zr-MOF to treat A549 tumor-bearing BALB/c nude mice via dual sensitization of RT (**Figure 1A**) 41. Zr-MOF consisting of Zr^4+^ as metal nodes and 1,4-benzenedicarboxylic acid (BDC) as organic linkers, was applied to load quercetin (QU) radiosensitizer with a loading amount of 20.7%, followed by the modification of bovine serum albumin (BSA) to develop Zr-MOF-QU with an average diameter of 62 nm. Experimental results demonstrated that Zr-MOF-QU could effectively inhibit the catalytic activity of carbonic anhydrase IX (CA IX) through the association of BDC decomposed from Zr-MOF-QU and the Zn^2+^ of CA IX in the TME, which was beneficial to alleviate the hypoxia-induced resistance at tumor sites, improving the RT therapeutic effect of the tumor. Moreover, owing to the good X-ray attenuation ability of QU in the Zr-MOF-QU, the as-prepared nanocomposite acted as a radiosensitizer to exhibit a preeminent RT sensitization effect both *in vitro* and *in vivo* via promoting the aggregation of γ-H_2_AX during DNA breakage. Therefore, Zr-MOF-QU, with good biodegradability, dual sensitization effect, and negligible side effects, possessed the ability to change the RT resistance in A549 tumor-bearing mice combined with RT.

Subsequently, Tang, Li, and coworkers fabricated a catalase-like MOF-based radiosensitizer, i.e., MnTCPP-Hf-FA NPs, to improve hypoxia-induced radioresistance (**Figure 1B**) 42. TCPP-Hf MOF were constructed from Hf clusters and tetrakis(4-carboxyphenyl) porphyrin (TCPP) as organic ligands by a solvothermal method. Then, Mn ions and folic acid (FA) were integrated into TCPP-Hf MOF through chelation interaction between Mn ions and TCPP and coordination between FA and Hf clusters to develop catalase-like MnTCPP-Hf-FA NPs equipped with good biocompatibility and tumor targeting capability. Owing to the presence of Hf clusters and Mn ions, the as-prepared MnTCPP-Hf-FA NPs could not only decompose endogenous H_2_O_2_ into O_2_ to enhance the RT effect of hypoxic tumors but also convert O_2_ and H_2_O into ROS to induce apoptosis of tumor cells, which was conducive to improve the therapeutic effect of hypoxic tumors. Besides, O_2_ and ROS could be rapidly diffused by the porous structure of MnTCPP-Hf-FA NPs. *In vivo* studies further proved that the MnTCPP-Hf-FA NPs could inhibit the growth and prevent the recurrence of hypoxic tumors under X-ray irradiation.

Recently, Tian, Bian, and coworkers designed a biodegradable “all-in-one” nanohybrid, namely UiO-66/Au-ASO/PEG (UAAP), to enhance the RT effect of triple-negative breast cancer by a dual exogenous/endogenous CA IX inhibition strategy (**Figure 1C**) 38. Au NPs regarded as radiosensitizers were modified on the surface of UiO-66 consisting of Zr^4+^ nodes and *p*-phthalic acid (PTA) ligands. After the decoration of thiolated CA IX antisense oligonucleotide (ASO) and polyethylene glycol (PEG) via Au-S bonds, the biodegradable UAAP was successfully prepared. In the TME, PTA degraded from UiO-66 could bind to Zn^2+^ of CA IX to inhibit the activity of CA IX, alleviating tumor hypoxia-induced resistance and improving the RT effect. Meanwhile, CA IX ASO, considered the endogenous inhibitory strategy, could knockdown the CA IX. Notably, in addition to providing binding sites for thiolated ASO and PEG, Au NPs also served as radiosensitizers to enhance the RT effect under X-ray irradiation. Such a nanohybrid showed high biocompatibility and exceptional tumor inhibition rate by alleviating the hypoxia-induced resistance and Au NPs-mediated radiosensitization in MDA-MB-231 tumor-bearing mice.

Very recently, Cheng and coworkers reported a nanoscale UiO-66-NH_2_(Hf) synthesized by a simple one-step strategy under atmospheric pressure for treating esophageal cancer by RT (**Figure 1D**) 43. Hf bearing high-Z element as the metal nodes and 2-amino-terephthalic acid (NH_2_-BDC) as the organic ligands were used to synthesize UiO-66-NH_2_(Hf) with a spherical shape and an average diameter of 95 ± 18 nm via a solvothermal method. Meanwhile, according to the results of nitrogen adsorption and desorption isotherm, the specific surface area and pore size of UiO-66-NH_2_(Hf) were determined as 415.5 m^2^g^-1^ and 0.6 nm, respectively, suggesting the porous nature and the permanent pore space of UiO-66-NH_2_(Hf). Notably, the good stability of UiO-66-NH_2_(Hf) in phosphate-buffered saline (PBS) and Dulbecco's modified Eagle's medium (DMEM) made it possible for biomedical application. *In vitro* studies showed that UiO-66-NH_2_(Hf) could effectively promote the absorption of X-ray to trigger DNA breakage and apoptosis of tumor cells under X-ray irradiation, thus enhancing the RT effect. In addition, *in vivo* experimental results further demonstrated the remarkable effect of UiO-66-NH_2_(Hf) as a radiosensitizer in tumor therapy, which provides a new way to fabricate MOF-based radiosensitizers for enhanced RT via a simple strategy.

### 2.2 Chemotherapy

Currently, chemotherapy is still one of the most effective and common methods for clinical tumor treatment 74. Chemotherapeutic drugs or prodrugs, such as doxorubicin (DOX), 5-fluorouracil (5-Fu), oxaliplatin (OxPt), and paracetamol (APAP), are widely used to treat various solid and non-solid tumors 75,76. However, the immediate use of chemotherapeutic drugs suffers from the drawbacks of low bioavailability, poor tumor permeability, easy generation of multidrug resistance, and uncontrolled side effects, seriously reducing the therapeutic efficacy 77. Nanoscale MOF-based nanoplatforms can encapsulate/anchor anticancer drugs/prodrugs to improve drug accumulation at tumor sites through the EPR effect. Besides, active targeting or functional entities can be decorated on the surface of MOFs through covalent and non-covalent interactions to increase the active targeting and long-circulating capabilities, which is beneficial for enhancing chemotherapy efficacy and reducing side effects 78,79.

**Figure 2:**
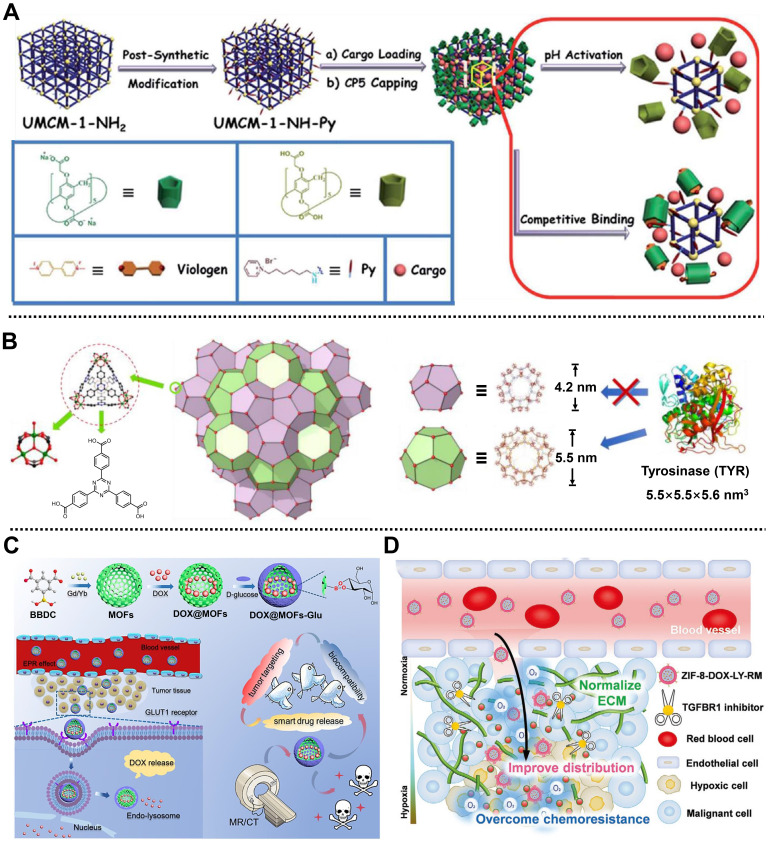
** (A)** Schematic diagram of the synthetic process and cargo release behavior of cargo loaded CP5-capped UMCM-1-NH-Py used in chemotherapy. Adapted with permission from 44. Copyright 2015, Royal Society of Chemistry. **(B)** The secondary building block of NPCN-333(Al) and two types of pores in it. Adapted with permission from 47. Copyright 2018, Wiley-VCH Verlag GmbH&Co. KGaA, Weinheim. **(C)** Schematic representation of the design of DOX@MOFs-Glu for imaging-guided chemotherapy. Adapted with permission from 48. Copyright 2019, American Chemical Society. **(D)** Diagram of the delivery and distribution at tumor sites of ZIF-8-DOX-LY-RM. Adapted with permission from 49. Copyright 2021, Wiley-VCH Verlag GmbH&Co. KGaA, Weinheim.

In 2015, in collaboration with Wang, we fabricated a dual-stimuli-responsive nanoplatform based on University of Michigan Crystalline Material (UMCM)-1-NH_2_ and carboxylatopillar[5]arene (CP5) nanovalves, namely CP5-capped UMCM-1-NH-Py, for the controlled release of cargo and tumor therapy (**Figure 2A**) 44. UMCM-1-NH-Py consisting of Zn^2+^ and NH_2_-BDC/4,4',4''-benzene-1,3,5-triyl-tribenzoic acid (BTB) ligands was used to load Rhodamine 6G or DOX and showed pH- and competitive agent-responsive release behavior due to the tunable host-guest interactions between CP5 nanovalves and the Py stalks onto the surface of UMCM-1-NH-Py. Moreover, this nanoplatform exhibited negligible premature release, low cytotoxicity, and good biodegradability and biocompatibility, which could be utilized as a smart drug delivery system for cell imaging and cancer therapy. Subsequently, a Zn^2+^ and thermal responsive nanocomposite based on positively charged quaternary ammonium (Q)-modified UiO-66-NH_2_ and negatively charged CP5 supramolecular nanovalves was designed for on-demand delivery of 5-Fu to achieve tumor therapy 45. UiO-66-NH_2_ consisting of NH_2_-BDC ligands and Zr_6_ clusters possessed high stability and large surface area, which was favorable for the efficient loading of 5-Fu. Under the pathological condition, a high concentration of Zn^2+^ weakened the binding affinity between CP5 and Q, leading to the separation of CP5 and Q to realize drug release. Meanwhile, external heating could also reduce the association intensity between CP5 and Q, enabling temperature-responsive drug release. Similarly, considering the high Ca^2+^ concentration in bone tumor cells, a Py-functionalized Zr-MOF modified with CP5 gatekeeper was prepared to treat bone tumors 46. Drug release results showed that the release of 5-Fu was precisely controlled under the stimulation of high Ca^2+^ concentration, low pH, and hyperthermia, thereby realizing the treatment of bone tumor-related diseases.

Interestingly, Zhou and coworkers developed a tyrosinase (TYR)-MOF employing NPCN-333(Al) as a potent prodrug activator for cancer therapy in HeLa tumor-bearing xenograft mice by tumor-specific prodrug activation 47. NPCN-333(Al) with a diameter of around 100 nm was constructed from trimeric-oxo clusters as metal nodes on the corner and 4,4',4''-s-triazine-2,4,6-triyl-tribenzoic acid (TATB) as ligands on the surface by solvothermal method. Due to the presence of the mesoporous cavity with a size of 5.5 nm in NPCN-333 (**Figure 2B**), the TYR was encapsulated and protected in its skeleton to form TYR-NPCN-333(Al) with good stability in an acidic microenvironment and proteolytic conditions. Through generating ROS and depleting glutathione (GSH), TYR-NPCN-333(Al) effectively activated the prodrug APAP into toxic 4-acetamido-o-benzoquinone in a long-lasting manner, thus inhibiting cell proliferation and causing apoptosis/necrosis at tumor sites. Therefore, TYR-NPCN-333(Al) nanoreactor with high enzyme encapsulation ability, good chemical stability, and facile post-functionalization possessed great potential in biomedical and biotechnological fields.

For another instance, Yin, Sun, and coworkers structured a smart MOF-based theranostic nanoplatform via a universal and facile strategy for targeting the release of DOX and imaging-guided precise chemotherapy of tumors (**Figure 2C**) 48. Gd/Yb as metal nodes and 5-boronobenzene-1,3-dicarboxylic acid (BBDC) as linker ligands were used to prepare Gd/Yb-MOFs. After the encapsulation of DOX, D-glucose as a pH-responsive gatekeeper and a targeting molecule was further coated on the outer surface of the nanoplatform via a reversible diol-borate condensation between D-glucose and boric acid groups in BBDC to develop DOX@MOFs-Glu with good biocompatibility and tumor targeting. Both *in vitro* and *in vivo* results indicated that DOX@MOFs-Glu possessed pH-responsive DOX release, good biocompatibility, active tumor-targeting, and imaging-guided tumor therapy capacities. Moreover, this convenient strategy for nanoplatform preparation could be easily applied to design and construct various nanoplatforms, such as Yb-MOF-Glu for gastrointestinal tract imaging, which paves the way for image-guided precise chemotherapy using smart MOF-based nanoplatforms.

Recently, Yang, Wang, Xia, and coworkers constructed a biomimetic MOF-based nanoplatform camouflaged by an erythrocyte membrane, namely ZIF-8-DOX-LY-RM, for alleviating hypoxia-mediated chemoresistance in 4T1 tumor-bearing mice 49. ZIF-8 composed of Zn^2+^ ions and 2-methylimidazole (2-MIM) ligands served as reservoirs to co-load anticancer drug DOX and type 1 transforming growth factor β receptor inhibitor LY364947 (LY) with high loading efficiency through *in situ* encapsulation, followed by the coating of red blood cell membrane on the outer surface to enhance the stability and tumor accumulation of the nanoplatform. The as-synthesized ZIF-8-DOX-LY-RM with a diameter of 125 nm exhibited pH-responsive drug release behavior and excellent stability in PBS and complete cell medium, which was conducive to further application of the nanoplatform in cancer therapy. In the TME, ZIF-8-DOX-LY-RM could effectively remove the extracellular matrix through the controllable release of LY, which increased the penetration of nanoplatform and generation of ROS, alleviating hypoxia-mediated chemoresistance of DOX (**Figure 2D**). As expected, ZIF-8-DOX-LY-RM, with good biosafety and enhanced distribution, demonstrated a high tumor inhibition rate *in vivo*, which opens an avenue to enhance the chemotherapy efficacy of solid tumors.

### 2.3 Chemodynamic therapy

In 2016, inspired by the Fenton reaction and Fenton-like reaction, CDT was proposed by Shi and Bu 80, and then widely used in treating diseases, especially tumor therapy. The mechanism of CDT is catalyzing endogenous substances H_2_O_2_ into highly cytotoxic •OH by metal ions, such as Fe^3+^, Cu^2+^, and Mn^2+^, followed by destroying the intracellular redox equilibrium to induce tumor cell death 2. Notably, the low acidity and high H_2_O_2_ content in the TME provide a suitable response environment for CDT-mediated tumor therapy. CDT possesses the advantages of independence of O_2_, external energy and equipment, high specificity, and low side effects on normal tissues; however, it suffers from the insufficiency of H_2_O_2_ content dependence, which may reduce the efficacy. Interestingly, MOFs composed of metal ions/clusters and organic ligands having tunable pore sizes, facile functionalization, good biocompatibility, and degradability can effectively improve the bottleneck faced in CDT. MOF-based nanoplatforms enhance the CDT effect against tumors by efficiently loading metal ions/NPs, generating metal ions by self-degradation, and increasing H_2_O_2_ content at tumor sites. In addition, metal ions (such as Fe^3+^, Cu^2+^, and Mn^2+^,) in MOFs endow MOF-based nanoplatforms with catalytic properties, depleting GSH, and imaging capabilities, which is conducive to enhancing the tumor therapeutic efficacy and realizing cancer theranostics 81.

**Figure 3:**
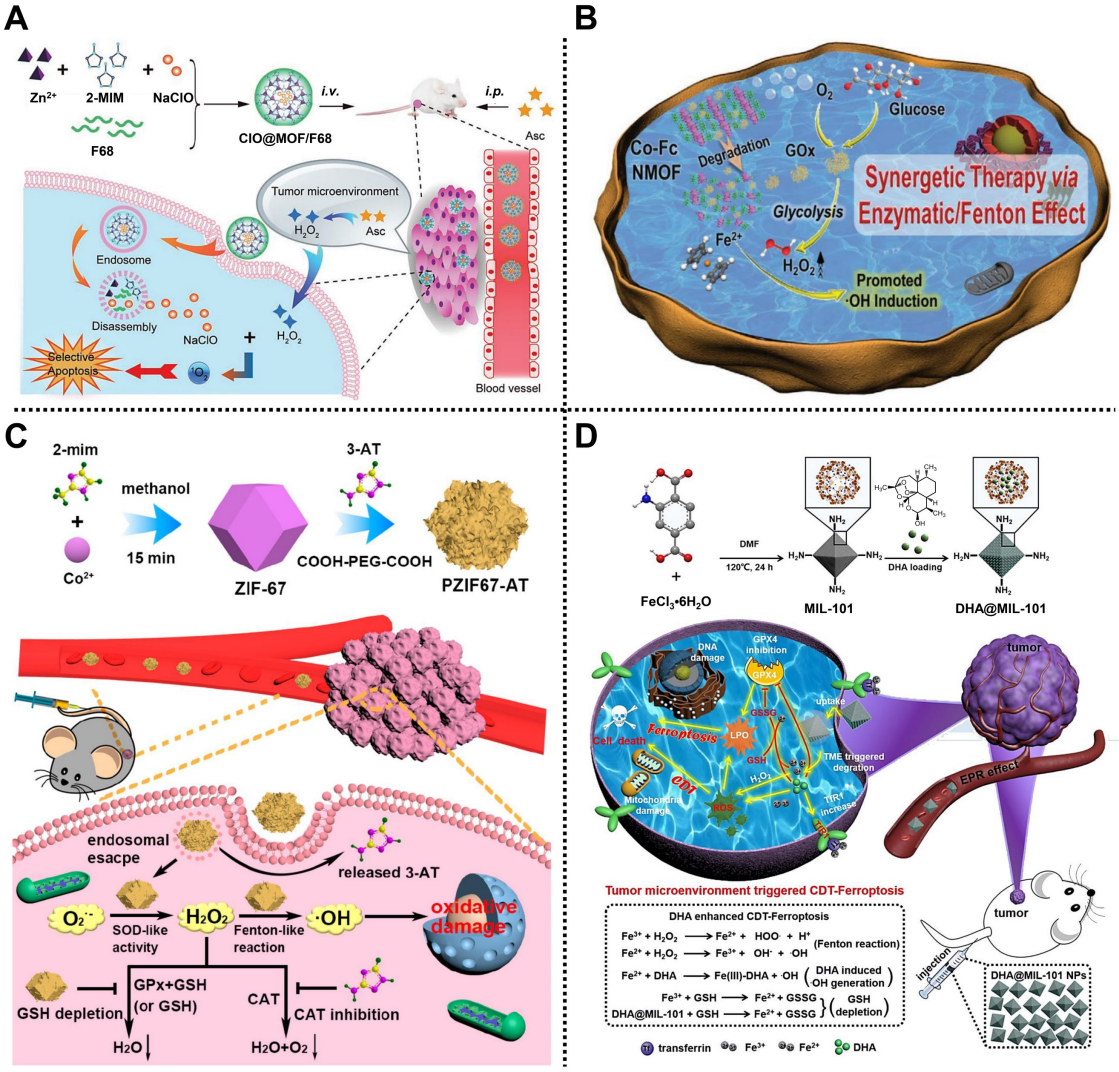
** (A)** Schematic illustration of the preparation of ClO@MOF/68 and its usage for treating mammary carcinoma by CDT. Adapted with permission from 50. Copyright 2019, Wiley-VCH Verlag GmbH&Co. KGaA, Weinheim. **(B)** The application of Co-Fc@GOx to induce CDT. Adapted with permission from 51. Copyright 2020, Wiley-VCH Verlag GmbH&Co. KGaA, Weinheim. **(C)** The fabrication of PZIF-67-AT and its mechanism for tumor therapy mediated by CDT. Adapted with permission from 52. Copyright 2020, American Chemical Society. **(D)** The synthesis of DHA@MIL-101 and its mechanism in CDT against tumor. Adapted with permission from 53. Copyright 2022, Springer Nature.

For example, in 2019, Zhao and coworkers manufactured an energy-free nanocarrier based on ZIF-8 and amphiphilic poloxamer 188 (F68) to create ClO@MOF/68 for treating mammary carcinoma via ^1^O_2_-based CDT (**Figure 3A**) 50. ZIF-8 was employed to load NaClO that could react with ascorbate to generate H_2_O_2_ for enhancing ^1^O_2_ at the tumor sites, and then capped with the F68 layer to form spherical ClO@MOF/68. Experimental results indicated that ClO@MOF/68 with good biocompatibility, high ClO^-^ loading, and pH/ROS-triggered degradability could accumulate at tumor sites through passive targeting after intravenous administration and possessed the ability to react with intraperitoneally injected ascorbate for the generation of ^1^O_2_, achieving tumor therapy in 4T1 tumor-bearing xenograft mice through ^1^O_2_-based CDT with negligible adverse effects. This work addressed the difficulty of ClO^-^ transport *in vivo* through constructing a MOF/polymer nanocarrier, revealing a new pathway for ROS-based selective CDT of tumors.

Subsequently, Han, Peng, Li, and coworkers reported a cascade Co-ferrocene (Fc) NMOF for promoting CDT in 4T1 tumor-bearing mice (**Figure 3B**) 51. Co-Fc@GOx with a diameter of ~80 nm was fabricated by loading GOx in Co-Fc NMOF consisting of Co^2+^ and Fc(COOH)_2_ as metal sources and organic ligands. Due to the existence of Fe^2+^ in the ferrocene ligands, Co-Fc@GOx showed high Fenton reaction ability. Under the TME, endogenous glucose and O_2_ could be efficiently catalyzed by GOx encapsulated in Co-Fc@GOx to generate gluconic acid and H_2_O_2_ via the glycolysis pathway, enhancing the acidity and the amount of H_2_O_2_. Simultaneously, the produced H_2_O_2_ was converted into toxic •OH by Co-Fc@GOx through the Fenton reaction, which greatly enhanced the effect of CDT. Experimental results demonstrated that Co-Fc@GOx with good degradability, remarkable glucose consumption, and high H_2_O_2_/•OH generation capacity was an outstanding nanoplatform for effective tumor growth inhibition ability via CDT.

In the same year, Qu, Ren, and coworkers designed a MOF-based H_2_O_2_ homeostasis disruptor, namely PZIF-67-AT, to elevate the intracellular H_2_O_2_ level by enhancing H_2_O_2_ generation and inhibiting H_2_O_2_ elimination for CDT of tumor (**Figure 3C**) 52. ZIF-67 constructed from cobalt ions and 2-MIM was modified with a small-molecule inhibitor called 3-amino-1,2,4-triazole (3-AT) by a post-synthetic modification strategy, reaching an approximate loading efficiency of 28.5%. Then, PEG was further decorated on the outer surface to improve biocompatibility and physiological stability. According to the flow cytometry and confocal laser scanning microscopy studies, the as-obtained PZIF-67-AT was efficiently internalized into cells and accumulated in acidic lysosomes, facilitating the release of 3-AT. Moreover, PZIF-67-AT could enhance the accumulation of H_2_O_2_ in tumor cells by promoting the conversion of superoxide anions into H_2_O_2_, suppressing the catalase activity and depletion of GSH. Importantly, the increased H_2_O_2_ could be converted into toxic •OH to trigger intracellular oxidative stress, thus enhancing the CDT effect of tumors, which was further verified *in vitro* and *in vivo*.

Recently, Li, Wen, and coworkers proposed a peroxidase-like nanoreactor based on MIL-101, i.e., DHA@MIL-101, for Lewis lung cancer therapy by an efficient CDT method (**Figure 3D**) 53. Chinese herbal monomer-dihydroartemisinin (DHA), a sesquiterpene lactone compound extracted from Artemisia annua, was embedded in the pores of MIL-101 constructed from Fe^3+^ and NH_2_-BDC ligands to form DHA@MIL-101 equipped with improved water solubility and biocompatibility, reaching the loading capacity efficiency of 20 wt% for DHA. In this system, Fe^3+^ released from DHA@MIL-101 by self-degradation in the TME could convert endogenous H_2_O_2_ into •OH through the Fenton reaction. Moreover, the released DHA not only served as an amplifier activated by iron ions to produce toxic •OH due to the existence of the unstable endoperoxide bridge (-O-O-) in its skeleton, but also produced ROS by recruiting an influx of iron ions. Besides, DHA could also direct the inhibition of the activity of GSH peroxidase 4, resulting in the apoptosis of the tumor. Importantly, both *in vitro* and *in vivo* results demonstrated the anticancer effect of DHA@MIL-101.

### 2.4 Phototherapy

#### 2.4.1 Photodynamic Therapy

PDT is a promising therapeutic strategy that utilizes photosensitizers in the presence of light and O_2_ to induce apoptosis and tissue damage 82. The mechanism of PDT contains two types: type Ⅰ PDT that generates ROS such as superoxide anion, •OH, and H_2_O_2_, as well as type Ⅱ PDT that generates highly cytotoxic ^1^O_2_ 83,84. During PDT, photosensitizers change from the ground state (S_0_) to the first excited state (S_1_) or the second excited state (S_2_) after absorbing the energy of the photon under light irradiation with the appropriate wavelength. S_2_ decays rapidly to S_1_ via internal conversion. Meanwhile, unstable S_1_ can be transformed into a more stable excited triplet state (T_1_) through the intersystem crossing. Notably, T_1_ can react with cellular substrates to form free radicals via an electron transfer mechanism, and the free radicals further react with O_2_ or H_2_O to generate ROS (such as •OH and O_2_^•-^), thus inducing cell apoptosis (type Ⅰ PDT process). In addition, the energy of T_1_ can be transferred to ^3^O_2_ to generate highly cytotoxic ^1^O_2_ (type Ⅱ PDT process) 85. Compared with traditional chemotherapy and RT, PDT exhibits the superiorities of fewer side effects, non-invasiveness, and high selectivity, which makes it widely applied in the clinical treatment of pancreatic cancer, skin cancer, head/neck cancer, etc 86,87. Despite considerable progress in cancer therapy by PDT, it still suffers from poor photosensitizer stability, limited irradiation depth, and high dependence on the O_2_ concentration 88. Intriguingly, due to the high porosity and facile functionalization, MOF-based nanoplatforms serve as nanocarriers to deliver photosensitizer molecules/NPs into tumor sites with high loading capability, distinctly increasing the stability/targeting of photosensitizers and improving the therapeutic performance. Moreover, smart MOFs constructed from porphyrin-based organic ligands directly act as photosensitizers for PDT, providing a promising option for PDT against tumors 89,90.

**Figure 4:**
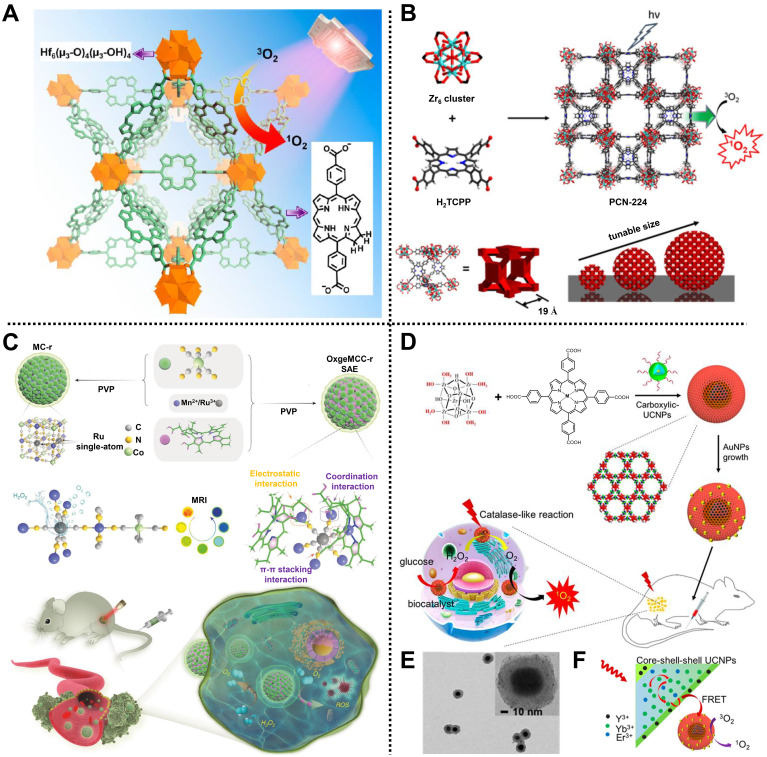
** (A)** Schematic representation of ^1^O_2_ generation by DBC-UiO under light irradiation. Adapted with permission from 55. Copyright 2015, American Chemistry Society. **(B)** The structure, diameters, and pore size of PCN-224 and its application in generating ^1^O_2_ for PDT. Adapted with permission from 56. Copyright 2016, American Chemical Society. **(C)** The construction of OxgeMCC-r SAE and its application in PDT against tumors. Adapted with permission from 58. Copyright 2020, Springer Nature.** (D)** The preparation of UMOFs@Au NPs and the application in PDT. **(E)** scanning electron microscopy (SEM) image of UMOFs@Au NPs and **(F)** its mechanism diagram of ^1^O_2_ generation under NIR laser irradiation. Adapted with permission from 59. Copyright 2020, American Chemical Society.

For instance, Lin and coworkers reported a nanoscale Hf-porphyrin MOF, namely DBP-UiO, for treating resistant head and neck cancer by PDT 54. Porous DBP-UiO synthesized by 5,15-di(*p*-benzoato)porphyrin (H_2_DBP) ligands and Hf^4+^ nodes via a solvothermal reaction was used as a highly effective photosensitizer with an average diameter of 76.3 nm. Due to the site isolation of H_2_DBP ligands and the inherent porous structure, DBP-UiO exhibited an excellent ^1^O_2_ generation efficiency. Moreover, *in vivo* study using DBP-UiO material showed high tumor suppression and negligible toxicity in SQ20B subcutaneous xenograft mice after PDT, indicating a great promise in resistance to cancer therapy. Similarly, a chlorin-based MOF with improved photophysical properties and higher ^1^O_2_ generation efficiency, consisting of Hf^4+^ metal ions and 5,15-di(*p*-benzoato)-chlorin (H_2_DBC) ligands, namely DBC-UiO, was further developed by the same group, demonstrating remarkable PDT effect in both CT26 and HT29 colorectal adenocarcinoma mouse models (**Figure 4A**) 55.

Interestingly, Zhou and coworkers synthesized a size-controllable Zr(IV)-based porphyrinic MOF modified with FA as an active targeting modality for PDT (**Figure 4B**) 56. Spherical PCN-224 comprised Zr_6_ as metal clusters and tetrakis(4-carboxyphenyl)porphyrin (H_2_TCPP) as ligands via a bottom-up method. Meanwhile, the regulated sizes of PCN-224 could be obtained by adjusting the concentrations of benzoic acid and H_2_TCPP, in which the 90 nm-PCN-224 showed the highest uptake amount and significant PDT effect in HeLa cells. In addition, FA was decorated on the surface of PCN-224 through the coordination between carboxylate groups of FA and Zr_6_ clusters to improve the active targeting of MOF, thus enhancing PDT efficacy.

Subsequently, Nie, Li, Zhang, and coworkers constructed a biomimetic and multifunctional MOF-based nanoparticle, namely aMMTm, to realize GSH scavenging and antiangiogenesis therapy in 4T1 tumor-bearing mice via PDT 57. Photosensitive porphyrinic Zr-MOF (PCN-224) was used to load Food and Drug Administration (FDA)-approved vascular endothelial growth factor receptor 2 (VEGFR2) inhibitor Apatinib, followed by the coating of MnO_2_ shell and cell membrane extracted from mouse breast cancer cell line 4T1 layer, in turn, to develop the core-shell nanocomposite aMMTm with an excellent encapsulation efficiency of >98.3% for Apatinib. In the TME, excessive GSH could be neutralized by the inner MnO_2_ shell on the surface of aMMTm, which was beneficial to blocking the PDT-induced angiogenesis process and triggering the efficient release of Apatinib. Besides, Mn^2+^ as the reaction product could be applied as a contrast agent for *in vivo* tumor magnetic resonance imaging (MRI). Importantly, owing to the modification of the cell membrane, aMMTm exhibited strong homologous targeting ability, high anticancer effect, and good biocompatibility both *in vitro* and *in vivo*.

For another instance, using an *in situ* one-pot multicomponent self-assembly strategy, Zhao, Guo, and coworkers designed a versatile MOF-based nanoagent, i.e., OxgeMCC-r SAE, to increase O_2_ generation at solid tumor sites for cancer therapy by PDT (**Figure 4C**) 58. Mn_3_[Co(CN)_6_]_2_ MOF consisting of Mn^2+^ and [Co(C≡N)_6_] organic ligands was used to anchor single-atom ruthenium (Ru) and accommodate hydrophobic photosensitizer chlorin e6 through coordination, hydrophobic, and electrostatic interactions, achieving the formation of near-globular OxgeMCC-r SAE with a high loading weight ratio of ~2.23 wt% for Ru and loading efficiency of 75.8% for chlorin e6. In the TME, Ru in OxgeMCC-r SAE as high catalytic sites could rapidly catalyze endogenous H_2_O_2_ into O_2_ to ameliorate tumor hypoxia without self-consumed or requiring external activation, thus improving the ROS level and enhancing PDT efficacy. Meanwhile, owing to the presence of Mn^2+^, OxgeMCC-r SAE also exhibited an excellent T_1_-weighted MRI effect. Notably, high tumor inhibition, good biocompatibility, and large tumor site accumulation capacities of OxgeMCC-r SAE were demonstrated *in vivo*, which further illustrated the operability of self-assembled nanoagents for cancer therapy via PDT.

Recently, Chen, Huang, Fan, and coworkers reported an effective core-shell nanoreactor based on MOFs, ultrasmall Au NPs, and upconversion nanoparticles (UCNPs), UMOFs@Au NPs, for cancer therapy via PDT in U87MG tumor-bearing mice (**Figure 4D**) 59. PCN-222 MOF composed of Zr clusters and Fe(III) *meso*-tetrakis(4-carboxyphenyl)porphine chloride (TCPP-Fe) ligands was introduced on the surface of carboxylic acid modified UCNPs by a “solvent-assisted self-assembly” method, followed by the decoration of ultrasmall Au NPs with a diameter of 2 nm onto PCN-222 via an *in situ* reduction method to form core-shell UMOFs@Au NPs (**Figure 4E**). In this design, UMOFs@Au NPs decomposed glucose into H_2_O_2_ and further catalyzed H_2_O_2_ to generate O_2_ at tumor sites under near-infrared (NIR) laser irradiation. Moreover, the visible light converted from the NIR laser by UCNP cores could stimulate the nanoreactor to continuously produce ^1^O_2_, realizing a cascade reaction for PDT (**Figure 4F**). Overall, this work provides a new avenue to construct MOF-based cascade catalytic nanoreactors for efficient tumor therapy via PDT.

As mentioned above, various MOF-based nanoplatforms for tumor therapy were designed and developed in recent years to improve the poor stability of photosensitizers, limited irradiation depth, and high dependence on O_2_ concentration during PDT. Notably, considering the short diffusion distance of ROS, reasonable control of sizes, porosities, and colloidal stability of MOF-based nanoplatforms can be applied to improve the therapeutic effect of PDT. The construction of degradable hollow MOF-based nanoplatforms to efficiently load photosensitizers and maintain high O_2_ yield is also an emerging approach. Additionally, employing a NIR-Ⅱ laser with high tissue penetration as the light source is another way to improve the efficacy of PDT. Importantly, the biosafety and biodegradability of MOF-based nanoplatforms need to be considered simultaneously.

#### 2.4.2. Photothermal therapy

As one of the approaches for phototherapy of malignant tumors, PTT can induce thermal ablation and necrotic death of tumor cells by converting light energy into hyperthermia with photothermal agents 91,92. Generally, light sources of PTT include the first NIR window (ranging from 750 to 1000 nm) and the second NIR window (ranging from 1000 to 1700 nm), due to the high tissue penetration depth and low biological cell and tissue absorption of NIR laser 93. Moreover, compared with normal cells, cancer cells are more sensitive to hyperthermia 94, which is beneficial for improving anticancer efficacy and reducing side effects. PTT has been widely used in cancer treatment due to its non-invasiveness, high controllability, O_2_ independence, and negligible side effects 95. However, low tumor specificity and confined tissue penetration limit the further development of PTT. Through rational design and integration, numerous nanomaterials have been fabricated to enhance the efficacy of PTT 12,35,96. Among them, MOF-based nanoplatforms possess an irreplaceable role during PTT due to their high loading and easy conjunction capabilities for photothermal agents.

**Figure 5:**
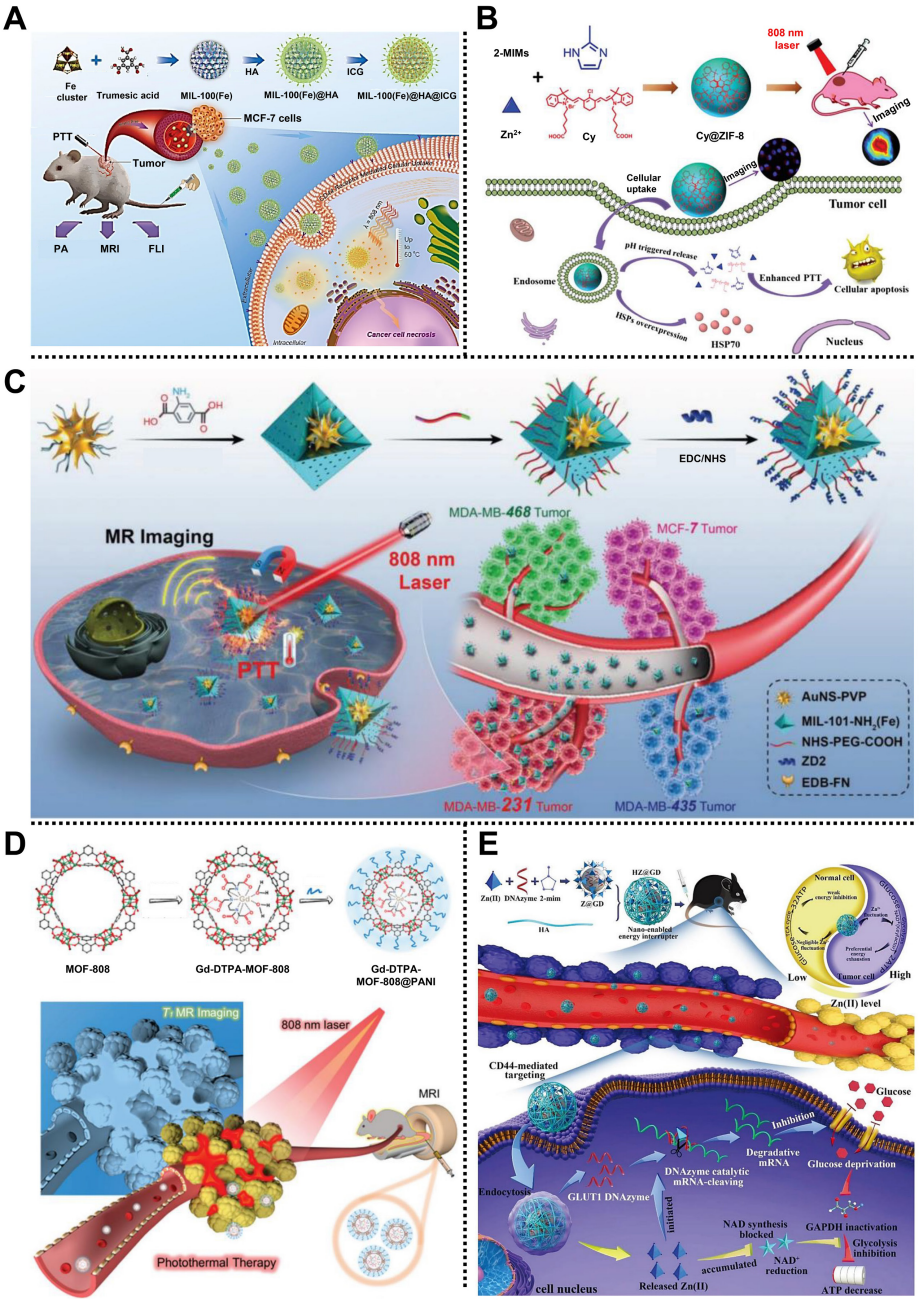
** (A)** Schematic diagram of the synthesis of MOF@HA@ICG NPs and the application for multimodal imaging-guided PTT. Adapted with permission from 60. Copyright 2017, American Chemical Society. **(B)** The preparation of Cy@ZIF-8 NPs and its usage for PTT of tumors. Adapted with permission from 62. Copyright 2018, Royal Society of Chemistry. **(C)** The fabrication of AuNS@MOF-ZD2 and the application for MRI imaging and PTT. Adapted with permission from 63. Copyright 2018, Wiley-VCH Verlag GmbH&Co. KGaA, Weinheim. **(D)** The synthesis of Gd-DTPA-MOF-808@PANI and its application in PTT. Adapted with permission from 64. Copyright 2021, Royal Society of Chemistry. **(E)** Schematic of the synthetic procedures of HZ@GD and the application to kill tumor cells via starvation therapy. Adapted with permission from 66. Copyright 2022, Wiley-VCH Verlag GmbH&Co. KGaA, Weinheim.

For example, Liu and coworkers prepared a multifunctional nanocomposite based on MIL-100(Fe) and hyaluronic acid (HA), that is, MOF@HA@ICG NPs, for multimodal imaging-guided PTT in MCF-7 tumor-bearing mice (**Figure 5A**) 60. MIL-100(Fe) constructed from Fe^3+^ nodes and 1,3,5-benzenetricaboxylic acid (BTC) organic ligands via hydrothermal reaction exhibited large pore sizes of 2.5 nm and 2.9 nm, which was suitable for cargo encapsulation. After conjugation with HA on the surface of MIL-100(Fe) to improve the targeting ability for CD44-overexpressed tumors, a NIR region organic dye indocyanine green (ICG), with poor water-solubility, was loaded to form MOF@HA@ICG NPs with a high loading capacity of 40%. Experimental results demonstrated that MOF@HA@ICG NPs with good biocompatibility, great cellular uptake, and photothermal stability effectively inhibited tumor growth by PTT under the guidance of fluorescence (FL) imaging, photoacoustic imaging (PAI), and T_2_-weight MRI.

Xie and coworkers prepared a MOFs@polymer nanocomposite named UiO-66@CyP through a universal and straightforward strategy to achieve the PTT of cancer 61. In this system, cyanine-containing polymer (CyP) was introduced onto the surface of UiO-66 by a multicomponent Passerini reaction to produce octahedral UiO-66@CyP with a homogeneous size of 100 nm and good dispersibility. The photothermal conversion efficiency of UiO-66@CyP was calculated as ∼27.3%, according to the experimental results. Under the 808 nm laser irradiation, UiO-66@CyP could effectively ablate tumor cells and inhibit tumor growth through PTT without apparent toxicity in CT26 tumor-bearing mice. This work paves a way to fabricate MOF@polymer nanocomposites for high-performance PTT of the tumor. Subsequently, the same research group designed and fabricated another MOF-based nanocomposite, namely Cy@ZIF-8 NPs, to encapsulate the NIR dye carboxyl-containing cyanine (Cy) for PTT of cervical carcinoma (**Figure 5B**) 62. Rhombic dodecahedral Cy@ZIF-8 NPs with an average size of 118.7 nm, pH-responsive ability, and good water solubility were constructed by a facile nanoprecipitation method. Due to the loading of Cy, Cy@ZIF-8 NPs exhibited excellent photothermal conversion efficiency of 33.2% and significant photostability upon 808 nm laser irradiation. Notably, both *in vitro* and *in vivo* studies demonstrated that Cy@ZIF-8 NPs possessed an outstanding ability to inhibit tumor growth with insignificant side effects of PTT, indicating the promising value of MOF in PTT.

Impressively, Zeng, Wu, Lu, and coworkers synthesized a smart core-shell nanoplatform based on gold nanostar (AuNS) and MIL-101-NH_2_(Fe), namely AuNS@MOF-ZD2, for T_1_-weighted MRI and targeting therapy of triple-negative breast cancer via PTT (**Figure 5C**) 63. Polyvinylpyrrolidone (PVP)-modified AuNS as the core was encapsulated by MIL-101-NH_2_ shell with four cycles by a step-by-step coating method, followed by the conjugation of NHS-PEG-COOH and short peptide (CTVRTSADC, ZD2) to develop AuNS@MOF-ZD2 with specific targeting capability and good biocompatibility. AuNS core endowed AuNS@MOF-ZD2 with an efficient T_1_-weighted MRI effect and photothermal conversion efficiency of 40.5%. Moreover, the decoration of the ZD2 on the surface of AuNS@MOF-ZD2 showed a specific targeting effect toward MDA-MB-231 cells rather than other subtypes of breast cancer cells, which was conducive to the precise therapy of breast cancer with molecular classification. AuNS@MOF-ZD2 with good biosafety exhibited excellent PTT effect, T_1_-weighted MRI, and specific targeting *in vivo*, which provides a new idea for visualization theranostics of different subtypes of breast cancers.

Recently, Yang, Lin, and coworkers fabricated a simple PTT nanoplatform based on MOF-808 and polyaniline (PANI), i.e., Gd-DTPA-MOF-808@PANI, for MRI-guided PTT in 4T1 tumor-bearing mice (**Figure 5D**) 64. MOF-808, consisting of Zr_6_O_4_-(OH)_4_(-CO_2_)_6_(HCOO)_6_ as clusters and BTC as ligands was used to restrict and graft the Gd chelate (Gd-DTPA) in its large cavity via a geometric confinement strategy, and then functionalized with PANI onto the surface to construct stable Gd-DTPA-MOF-808@PANI with high MRI relaxivity and good photothermal conversion efficiency of 30.6%. According to the results both *in vitro* and *in vivo*, Gd-DTPA-MOF-808@PANI displayed a strong T_1_-weighted MRI effect (5.4 times higher than that of commercial contrast Magnevist), remarkable PTT ability under 808 nm laser irradiation, and negligible side effects in 4T1 tumor-bearing mice, which makes MOF-based nanocomposites an appropriate candidate for cancer theranostics.

### 2.5 Starvation therapy

Compared with normal tissue, tumor cells require a large amount of nutrients and energy to maintain their survival and growth due to their disordered metabolic pathways. According to the Warburg effect, the glycolytic pathway is the primary energy source for tumor cells, making tumor cells consume more glucose than normal cells 97. Thus, Once the glucose supply is cut off, cancer cells will be “starved to death” 98. Starvation therapy has recently been considered a promising method that reduces necessary components for tumor growth. Glucose oxidase (GOx), a typical and natural glucose-consuming enzyme, has been extensively applied in the starvation therapy of tumors 99. However, GOx suffers from disadvantages of poor stability, low environmental tolerance, and easy variability, bringing about the reduced effect of starvation therapy. Notably, encapsulating or anchoring GOx in nanomaterials is expected to alleviate the abovementioned drawbacks. In the last few years, MOF-based nanocomposites, one of the most representative nanomaterials, have generated broad interest, accelerating the architecture of multifunctional nanovehicles to improve the starvation therapy effect 100,101.

For instance, Qu, Ren, and coworkers designed a biomimetic MOF-based nanocomposite, i.e., TGZ@eM, to co-load prodrug tirapazamine (TPZ) and GOx for starvation-activated colon cancer therapy in CT26 tumor-bearing mice 65. Acid-degradable ZIF-8 composed of Zn^2+^ and 2-MIM ligands was applied to co-load GOx and TPZ through a simple one-pot approach to obtain GOx and TPZ-loaded ZIF-8 (denoted as TGZ), further camouflaged by erythrocyte membrane for fabricating core-shell spherical TGZ@eM bearing good immunity-escaping and prolonged blood circulation features. In the TME, endogenous glucose and O_2_ could be consumed by GOx that released from TGZ@eM to trigger starvation and aggravate hypoxia in the tumor, which helped to effectively initiate the conversion of prodrug TPZ into highly toxic radicals, thereby inducing cell apoptosis. Both *in vitro* and *in vivo* proved the enhanced anticancer ability of TGZ@eM via starvation therapy in CT26 cells/CT26 tumor-bearing mice.

Interestingly, Shi, Liu, Zhang, and coworkers fabricated a pH-responsive degradable nanoplatform based on ZIF-8, i.e., HZ@GD, for the treatment of melanoma in B16-F10 tumor-bearing C57BL/6 mice (**Figure 5E**) 66. In this system, glucose transporters 1 (GLUT1) mRNA-cleaving DNAzyme (GD) was encapsulated in ZIF-8 with a high loading efficiency of ~81.5%, followed by the decoration of HA to enhance the tumor targeting of the as-prepared HZ@GD. Benefiting from the degradability in the acidic environment of tumors, ZIF-8, consisting of Zn^2+^ nodes and 2-MIM ligand, was capable of simultaneously releasing Zn^2+^ and GD into the cytoplasm. Subsequently, the released Zn^2+^ could not only trigger the decrease of NAD^+^ and the inactivation of GAPDH to achieve effective glycolysis inhibition but also active GD to downregulate the expression of GLUT1, which was beneficial to inhibiting the glycolysis process and achieving energy exhaustion in the tumor sites, providing a new paradigm for MOF-based nanocarrier to inhibit tumor growth via starvation therapy.

### 2.6 Immunotherapy

As a particular treatment method, immunotherapy triggers or enhances the defenses against tumor cells using own immune system of patients 102. Compared with traditional treatment methods, immunotherapy can guide the immune system to target tumor cells, which helps to reduce side effects. Meanwhile, immunotherapy has the advantages of wide adaptability and memory effect. Generally, various cells such as macrophages, natural killer cells, and T cells, hydrolase, defensin, and cytokines, including interleukin-1 (IL-1), interleukin-6 (IL-6), interferon-γ (IFN-γ), and tumor necrosis factor-α (TNF-α) may participate in the immunotherapy process 103. Immunotherapy provides a promising strategy for treating tumors and significant progress has been received. Still, some drawbacks, such as inflammatory response, complex immunosuppression TME, and atypical clinical reaction rates, limit its further application in tumor therapy 11,104. Due to the high loading capability, suitable size, diverse composition, tunable pore size, and EPR effect of MOFs, they are applied as nanocarriers in immunotherapy to load therapeutic agents, such as antigens, adjuvants, and immunomodulators, etc., for enhancing the therapeutic effect.

**Figure 6:**
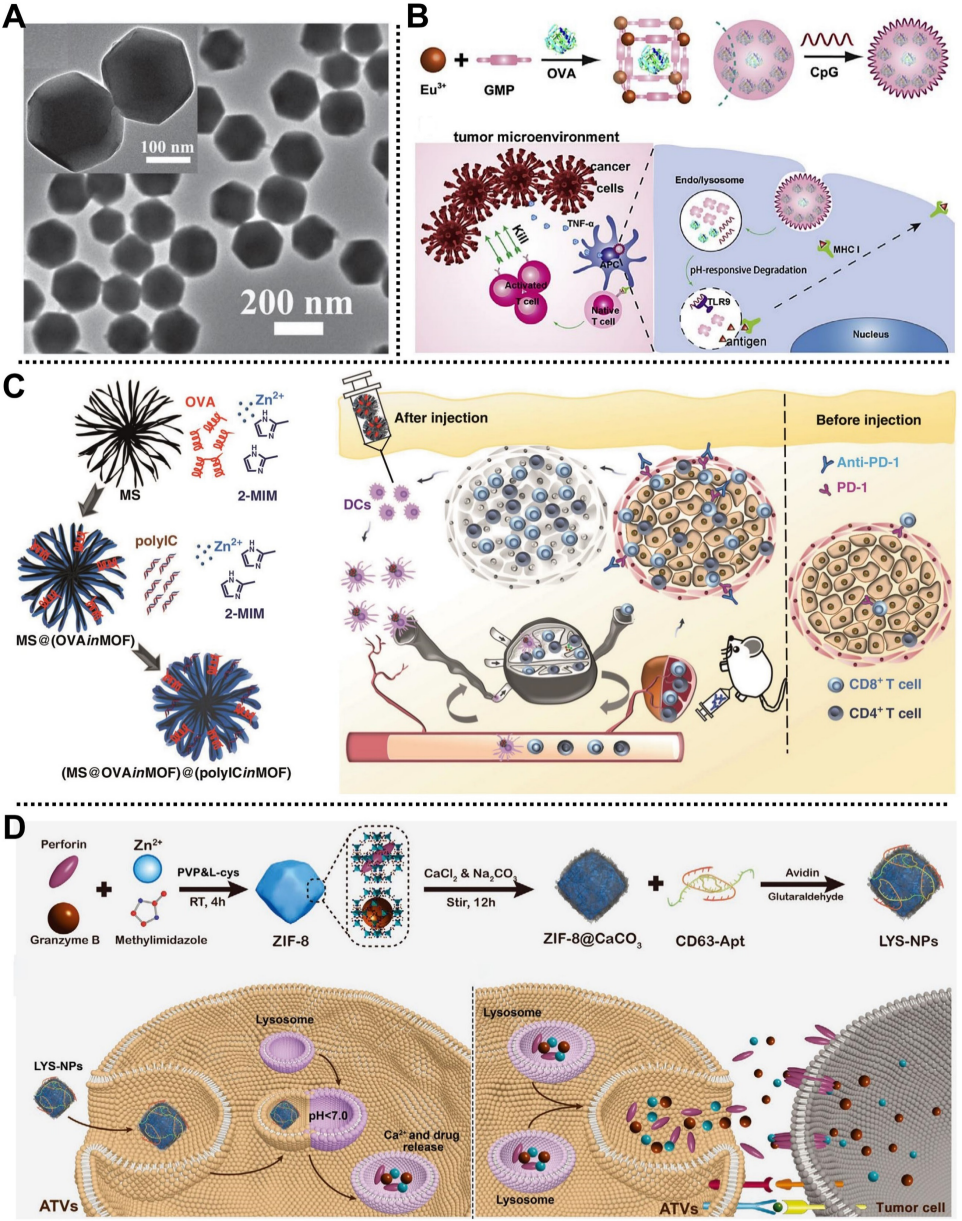
** (A)** Transmission electron microscope (TEM) image of OVA@ZIF-8. Adapted with permission from 67. Copyright 2016, Wiley-VCH Verlag GmbH&Co. KGaA, Weinheim. **(B)** Scheme of MOF-OVA@CpG for immunotherapy. Adapted with permission from 68. Copyright 2017, Elsevier. **(C)** Schematic description of the construction of MOF-gated MS and the application for cancer immunotherapy. Adapted with permission from 69. Copyright 2020, Springer Nature. **(D)** Schematic illustration of the fabrication of LYS-NPs and its application for cancer immunotherapy in 4T1-tumor-bearing mice. Adapted with permission from 70. Copyright 2021, Wiley-VCH Verlag GmbH&Co. KGaA, Weinheim.

In 2016, Qu, Ren, and coworkers fabricated a nanoscale MOF-based vaccine, namely OVA@ZIF-8-CpG, for cancer immunotherapy by eliciting strong humoral, cellular immune, and strong immune memory responses 67. Ovalbumin (OVA) as a model antigen was embedded into ZIF-8 to develop OVA@ZIF-8 with a diameter of 200 nm and high cellular uptake efficiency (**Figure 6A**), and then a negatively charged unmethylated cytosine-phosphate-guanine oligodeoxynucleotides (CpG ODNs) commonly applied as the vaccine adjuvant was modified onto OVA@ZIF-8 surface through electrostatic interaction to prepare OVA@ZIF-8-CpG bearing pH-responsive degradation ability, good biocompatibility, and strong immunogenicity. Notably, both *in vitro* and *in vivo* studies indicated that this dual-loading strategy allowed the as-prepared OVA@ZIF-8-CpG to deliver CpG ODNs and OVA antigen to the same antigen-presenting cells and release them at low pH conditions, which was conducive to inducing strong immune response including humoral and cellular immunes and robust immune memory response upon second exposure to the same antigen.

Similarly, Zhang, Li, and coworkers fabricated a simple pH-responsive nanoplatform for enhanced cancer immunotherapy in the B16-OVA melanoma cancer model (**Figure 6B**) 68. OVA antigen was loaded in the MOF consisting of Eu^3+^ as metal nodes and guanine monophosphate (GMP) as linkers by a one-pot synthetic method, followed by the introduction of CPG on the outer surface via Watson-Crick base pairing. Experimental results demonstrated that the as-obtained degradable nanocomposite possessed high OVA antigen encapsulation efficiency (55%, w/w), enhanced endo/lysosomal escape ability, accelerated CD8^+^ cytotoxic T lymphocytes activation, and improved anticancer effect (about 100% survival), paving the way for inducing robust immune responses for tumor therapy. Subsequently, Wang and coworkers prepared a core-shell NPs based on MOF-gated mesoporous silica (MS) to encapsulate OVA and immunopotentiator (polyinosinic-polycytidylic acid, polyIC) through a universal self-assembly route for immunotherapy in E.G7-OVA tumor-bearing mice (**Figure 6C**). As a result, the core-shell NPs exhibited strong antigen-specific immune responses and durable tumor suppression, according to experimental studies 69.

Recently, Zhang and coworkers fabricated a lysosome-targeting nanoplatform based on ZIF-8, i.e., LYS-NPs, for tumor therapy by immunotherapy in 4T1-tumor-bearing mice (**Figure 6D**) 70. In this system, therapeutic proteins, including perforin and granzyme B that could efficiently lyse tumor cells and promote tumor cell apoptosis, were encapsulated in acid-degradable ZIF-8 through a co-precipitation method. Subsequently, Ca^2+^ was deposited on the surface or inside of ZIF-8 to improve the biocompatibility of nanoplatform by mineralization and enhance the function of perforin and granzyme B. Moreover, CD63-aptamer was decorated on the outermost layer of the nanocarrier to form LYS-NPs with enhanced lysosome targeting T cells. LYS-NPs were degraded under the acidic conditions of the T cell lysosome, and then they released perforin, granzyme B, and Ca^2+^ were stored in the lysosome of T cells. Notably, once the major histocompatibility complex of the tumor cell activated the T cell receptor, the contents stored in the T cell lysosomes were rapidly released into immunological synapses to realize the immunotherapy of the tumor, which was proved by *in vitro* and *in vivo* studies.

## 3. Combination therapy

Single therapeutic strategies employing MOF-based nanoplatforms have achieved positive treatment effects, demonstrating their attractive advantages in cancer therapy. However, several intrinsic drawbacks of single therapy are still unavoidable 105. For example, although RT and chemotherapy have many applications and enhanced tumor eradication abilities, they suffer from severe side effects. CDT and starvation therapy possess the advantages of non-invasiveness and high selectivity but still face the shortcomings of H_2_O_2_ and O_2_ concentration dependence in the TME. Non-invasive and highly controlled phototherapy has the problem of restricted light penetration depth. Immunotherapy possesses broad applicability while exhibiting high inflammatory response and atypical clinical reaction rates. Compared with the single therapy methods, integrating two or more therapy methods in one system for synergistic tumor therapy can achieve a “1+1>2” cumulative effect, reduced therapeutic agent dosage, and low side effects 77. Generally, combination therapy possesses better therapeutic effects than single therapy in preclinical and clinical studies and can break through the limitations of single therapy 106,107. Therefore, there is an exciting opportunity to integrate multiple therapy methods into one MOF-based nanoplatforms for precision tumor therapy. Recently, various combination therapy incorporating MOF-based nanoplatforms have been developed to achieve superior antitumor effects 108,109. In this section, we summarize the recent representative MOF-based nanoplatforms in precision tumor therapy using bimodal therapy and multimodal therapy strategies (**Table 3**).

### 3.1 Bimodal therapy based on MOFs

#### Chemotherapy/CDT

**Figure 7:**
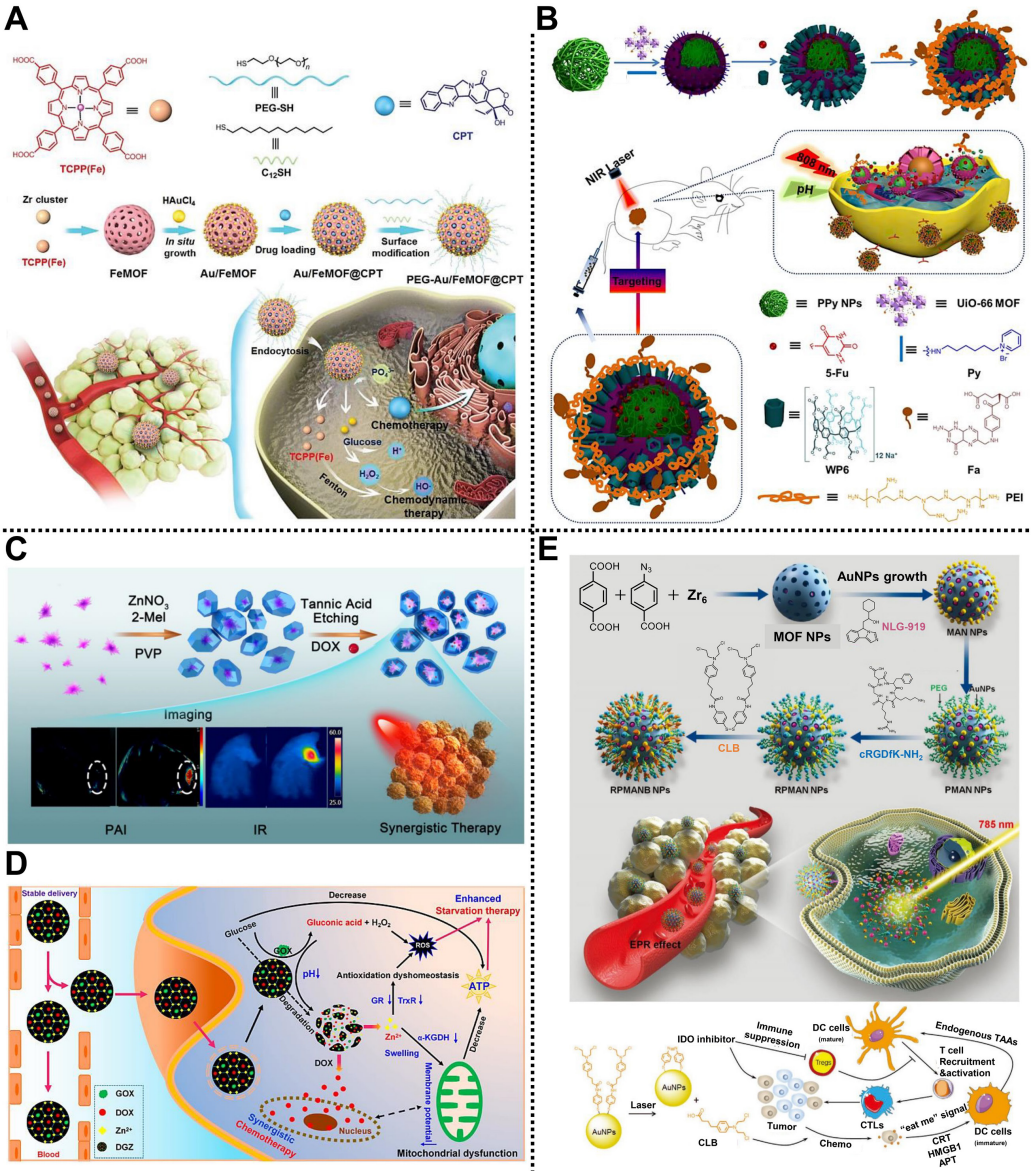
** (A)** Schematic diagram of the synthetic route of Au/FeMOF@CPT and its usage for synergistic chemotherapy and CDT. Adapted with permission from 110. Copyright 2020, Wiley-VCH Verlag GmbH&Co. KGaA, Weinheim. **(B)** The synthesis of multi-stimuli-responsive PUWPFa NPs and their application in tumor therapy by chemotherapy and PTT. Adapted with permission from 40. Copyright 2018, American Chemical Society. **(C)** The preparation of yolk-shell Au@MOF-DOX for imaging-guided chemotherapy and PTT. Adapted with permission from 112. Copyright 2019, American Chemical Society. **(D)** The mechanism of DGZ NPs for triple-negative breast tumor treatment by chemo-starvation therapy. Adapted with permission from 114. Copyright 2022, American Chemical Society. **(E)** The formation of RPMANB NPs and its simplified mechanism for treating tumors through chemo-immunotherapy. Adapted with permission from 115. Copyright 2021, Wiley-VCH Verlag GmbH&Co. KGaA, Weinheim.

Chemotherapy and CDT exhibit an excellent synergistic effect in inhibiting tumor growth. For instance, Wang, Mao, Yan, and coworkers prepared a smart nanocomposite based on MOFs and Au NPs for synergistic chemotherapy and CDT in HepG2 tumor-bearing nude mice (**Figure 7A**) 110. TCPP-Fe as organic ligands and Zr^4+^ as metal clusters were used to construct the FeMOF reservoir through a solvothermal strategy, followed by the in-situ growth of Au NPs on the surface of FeMOF. After loading the hydrophobic camptothecin (CPT), 1-dodecanethiol and PEG containing thiol were further modified by Au-S bond to develop PEG-Au/FeMOF@CPT with improved colloidal stability and blocking catalytic capability. In the TME, a high concentration of phosphate could effectively trigger the complete decomposition of the nanocomposite, promoting the release of CPT and chemotherapy. Moreover, H_2_O_2_ produced from glucose that was catalyzed by Au NPs was further converted into highly toxic •OH via the Fenton reaction for CDT. *In vitro* and *in vivo* studies showed that the nanocomposite possessed high tumor accumulation, negligible systemic toxicity, and good antitumor effect, providing new thinking for cancer therapy.

#### Chemotherapy/PTT

The cooperation of PTT and chemotherapy can promote the release of anticancer drugs through the thermal effect of PTT, increasing chemotherapy toxicity and achieving a synergistic effect. In 2018, we reported a multi-stimuli-responsive supramolecular nanocomposite with active targeting capability for synergistic chemotherapy and PTT of cervical cancer in HeLa tumor-bearing nude mice (**Figure 7B**) 40. Polypyrrole NPs with good photothermal conversion capability as the core was encapsulated by a UiO-66-NH_2_ shell, followed by the capping of water-soluble pillar[6]arene nanovalves to form PUWPFa NPs with multi-stimuli responsiveness on pH, temperature, and NIR light. After the encapsulation of 5-Fu, FA-modified polyethyleneimine as an active targeting antenna was coated on the outer layer of supramolecular nanocomposite through electrostatic interaction to enhance the tumor targeting and improve the therapeutic effect. Importantly, high photothermal conversion efficiency, controllable drug release, and efficient synergistic therapy effect for cervical cancer with negligible side effects were observed both *in vitro* and *in vivo*. Subsequently, another smart nanoplatform called AuMC NPs constructed from MIL-101(Fe)-NH_2_ shell, gold nanorods (AuNRs), and CP5 nanogates were structured to load 5-Fu for computed tomography (CT) imaging-guided cancer therapy, exhibiting multi-stimuli-responsive drug release by stimulants of pH, Ca^2+^, and hyperthermia, with high photothermal efficiency (38.69%), large 5-Fu loading capacity (193mg/g), and CT-guided synergistic anticancer efficiency 111.

Interestingly, Lin, Cheng, and coworkers designed a stimuli-responsive yolk-shell nanoplatform for imaging-guided tumor therapy via chemotherapy and PTT in H22 tumor-bearing mice (**Figure 7C**) 112. Star-shaped Au NPs acted as the NIR-Ⅱ photothermal yolks were coated with ZIF-8 shells, followed by loading of DOX to establish Au@MOF-DOX with a high photothermal conversion efficiency of 30.2% and loading ratio of 29% for DOX, which improved the efficiency of chemotherapy and PTT. Meanwhile, owing to the pH sensitivity of the ZIF-8 shell and the strong NIR-Ⅱ absorbance feature of the star-shaped Au yolk, Au@MOF-DOX was capable of releasing DOX on demand and infrared photothermal imaging /PAI properties in the TME. Upon the NIR-Ⅱ laser irradiation, the as-designed Au@MOF-DOX exhibited good biocompatibility and practical anticancer effect both *in vitro* and *in vivo*. This work paves the way to build MOF-based nanocomposites integrated with multifunctional NPs for cancer therapy.

#### Chemotherapy/PDT

ROS generated by PDT can promote the accumulation of anticancer drugs in tumor cells. Because of this, Liu, Ouyang, Li, and coworkers devised an intelligent nanohybrid based on UiO-66-NH_2_ for chemotherapy and PDT in HeLa tumor-bearing mice 113. After covalently binding 5-carboxyl fluorescein (5-FAM) on the surface of UiO-66-NH_2_, photosensitizer protoporphyrin precursor 5-aminolevulinic acid (5-ALA) was loaded in the pores of MOF reservoir, followed by the modification of pemetrexed (MTA, multi-target FA antagonist) through high affinity between MTA and unsaturated Zr on the surface of UiO-66-NH_2_ to form ALA@UiO-66NH-FAM@CP1 with high loading rates of 41.03 and 10.43 wt% for MTA and ALA, respectively. Meanwhile, 5-ALA and 5-FAM endowed the nanohybrid with PDT and FL imaging properties, respectively. In addition, according to the *in vitro* and *in vivo* experimental results, the as-prepared nanohybrid showed good biocompatibility, targeted delivery, and excellent anticancer effect via synergistic chemo-photodynamic therapy under 660 nm NIR irradiation.

#### Chemo/starvation therapy

Huang and coworkers engineered a smart MOF-based nanoreactor to encapsulate GOx and DOX for enhanced chemo-starvation therapy of tumor in the triple-negative breast tumor 114. ZIF-8 was used as a reservoir for loading GOx and anticancer drug DOX through a one-step self-assembly method to construct DGZ NPs with a diameter of 115 nm and a relatively coarse surface. Due to the presence of GOx, DGZ could catalyze glucose into gluconic acid and H_2_O_2_, causing a decrease in pH and an elevation in ROS. Meanwhile, the decreased pH led to the decomposition of DGZ and controllable release of DOX and Zn^2+^. As a result, the released Zn^2+^ could not only inactivate mitochondria in tumor cells by inhibiting oxidative respiration from inducing mitochondrial swelling and loss of membrane potential but also destroy the antioxidant system of tumor cells via inhibiting the activities of thioredoxin reductase and GSH reductase (**Figure 7D**). Importantly, the decreased glucose and ATP in tumor cells facilitated DGZ-mediated starvation therapy. This work effectively improved the synergistic chemo-starvation therapy efficiency by the decomposition components of DGZ, opening a new perspective for the treatment of tumors with MOF-based nanocomposites.

#### Chemo/immunotherapy

Wang, Mao, and coworkers reported an intelligent nanohybrid based on MOFs and Au NPs, namely RPMANB NPs, for tumor therapy through integrating activatable chemotherapy and immunotherapy in 4T1-tumor-bearing mice (**Figure 7E**) 115. Nanoscale UiO-66 MOF constructed from Zr^4+^ metal nodes and BDC/4-azidobenzoic acid (BCN3) ligands was used to load hydrophobic indoleamine 2,3-dioxygenase inhibitor (NLG919) with an encapsulation capacity of 8.7 ± 0.9%, followed by the anchoring of Au NPs on the surface of MOF via Au-thiol interaction. Subsequently, dibenzocyclooctyne-PEG-NHS (DBCO-PEG-NHS) and cyclo(Arg-Gly-Asp-D-Phe-Lys) (cRGDfK-NH_2_) components were decorated on the surface of MOF through copper-free click reaction and amine-NHS reaction, respectively, which enhanced the dispersity, stability, and active targeting of the as-obtained nanohybrid. Furthermore, chlorambucil-based chemotherapeutic prodrug (CLB) was further conjugated onto the surface of Au NPs by strong Au-thiol interaction, obtaining a loading capacity of 7.4 ± 0.6%. Interestingly, high-concentrated phosphate in tumor cells could disintegrate the structure of MOF to achieve explosive NLG919 release, which effectively inhibited the activity of IDO and overcame the immunosuppressive in the TME. Meanwhile, the CLB prodrug could be activated upon the NIR laser irradiation to induce immunogenic cell death. Synergistic chemo-immunotherapy investigations indicated that the nanohybrid could achieve effective tumor suppression by promoting the accumulation of cytotoxic T cells at tumor sites and suppressing regulatory T cells.

#### Chemoradiotherapy

**Figure 8:**
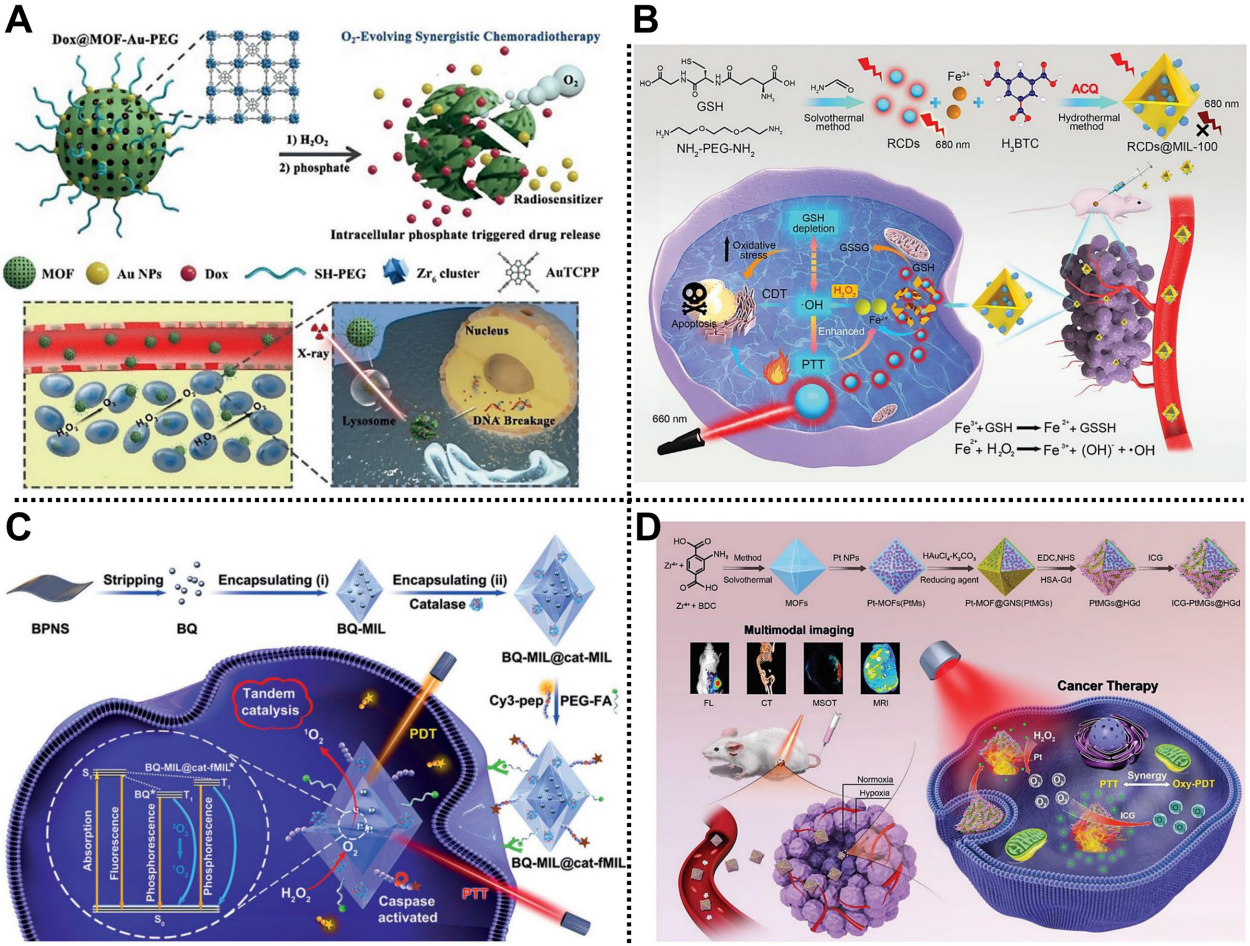
** (A)** Schematic illustration of the structure of Dox@MOF-Au-PEG and its application in synergistic chemoradiotherapy of tumors. Adapted with permission from 116. Copyright 2019, Wiley-VCH Verlag GmbH&Co. KGaA, Weinheim. **(B)** The construction of RCDs@MIL-100 and the application for imaging-guided CDT and PTT. Adapted with permission from 117. Copyright 2022, Wiley-VCH Verlag GmbH&Co. KGaA, Weinheim. **(C)** The principle of BQ-MIL@cat-fMIL for PTT and PDT of the tumor under NIR laser irradiation. Adapted with permission from 119. Copyright 2019, Wiley-VCH Verlag GmbH&Co. KGaA, Weinheim. **(D)** The ICG-PtMGs@HGd as smart nanoplatforms for multimodal imaging-guided PTT and PDT of tumors. Adapted with permission from 120. Copyright 2020, Wiley-VCH Verlag GmbH&Co. KGaA, Weinheim.

The combined use of chemotherapy and RT has a long history in preclinical and clinical tumor therapy applications. For example, Chen, Dai, Yu, Zhu, and coworkers designed an O_2_ self-supplying nanocomposite based on MOFs and Au NPs, namely Dox@MOF-Au-PEG, for synergistic chemoradiotherapy in U87MG tumor-bearing mice (**Figure 8A**) 116. Au NPs were modified on the surface of the porphyrinic MOF scaffold via an *in situ* growth method to construct a nanohybrid reservoir for anticancer drug DOX loading. Subsequently, the PEG layer was further decorated on the outer surface of the as-prepared nanocomposite to improve its colloidal stability and blood circulation time, which was beneficial to enhancing the accumulation of the nanocomposite at the tumor sites through the EPR effect. Moreover, the modification of Au NPs not only endowed the nanocomposite with the advantage of radiosensitization but also ensured excellent stability of the nanocomposite under high phosphate conditions. Interestingly, the as-prepared nanocomposite could catalyze H_2_O_2_ into O_2_ in the TME, improving O_2_-dependent RT. Both *in vitro* and *in vivo* experimental results demonstrated that Dox@MOF-Au-PEG possessed stimuli-responsive O_2_ generation, controllable DOX release, alleviation of tumor hypoxia, and excellent anticancer effects with negligible systematic toxicity, paving the way for the application of MOF-based nanocomposites in synergistic chemoradiotherapy of tumor.

#### CDT/PTT

The heat generated during PTT can accelerate the rate of the Fenton/Fenton-like reactions to enhance the production of ROS. Therefore, it is of great significance to realize the synergistic treatment of tumors using CDT and PTT 129. Liang, Zhao, Zhang, and coworkers reported a smart MOFs-based nanoplatform for tumor treatment via imaging-guided CDT and PTT (**Figure 8B**) 117. NIR emission carbon dots (RCDs) prepared by GSH and NH_2_-PEG-NH_2_ precursor were encapsulated by MIL-100(Fe) composed of Fe^3+^ nodes and BTC ligands through a one-pot hydrothermal method to form RCDs@MIL-100 with good physiological stability and aqueous dispersity. RCDs@MIL-100 exhibited no FL emission state due to the aggregation-caused quenching features of RCDs. However, high concentrations of GSH in the TME could reduce Fe^3+^ in the MIL-100(Fe) skeleton to Fe^2+^, which triggered GSH consumption, RCDs@MIL-100 decomposition, and RCDs release, achieving the FL recovery of RCDs and tumor imaging. Moreover, the NIR absorption property of RCDs endowed RCDs@MIL-100 with a high photothermal conversion efficiency of 31.2% and efficient PTT ability. Furthermore, the generated Fe^2+^ could convert H_2_O_2_ into highly toxic •OH through the Fenton reaction to realize CDT of tumors. This work provides an effective way to develop a promising MOF-based nanoplatform for cancer therapy.

#### PDT/starvation therapy

Zhang and coworkers designed a biomimetic MOF-based nanoreactor for cancer therapy via PDT and starvation therapy in 4T1 tumor-bearing mice 118. In this system, PCN-224 as the photosensitizer was used to co-load GOx and catalase on its surface through electrostatic interactions and further coated with 4T1 cancer cell membrane fragments to develop core-shell mCGP with good stability. In the TME, the as-fabricated mCGP could alleviate tumor hypoxia by catalyzing endogenous H_2_O_2_ to generate O_2_ and cut off the glucose metabolism in tumor cells by decomposing glucose into H_2_O_2_. Besides, the generated O_2_ could be converted into highly toxic ^1^O_2_ by PCN-224 shell under NIR laser irradiation to improve tumor therapy efficiency. The as-fabricated mCGP possessed immune escape and homotypic targeting ability, high tumor accumulation, and enhanced anticancer effect via PDT and starvation therapy, which provides a way to design and construct MOF-based nanoplatforms with cascade reaction ability for improving anticancer efficiency.

#### PTT/PDT

PDT and PTT are two non-invasive phototherapy methods, and their cooperation can achieve an enhanced anticancer effect. In 2019, Lei and coworkers fabricated a multifunctional nanohybrid based on MIL-101 and black phosphorus quantum dot (BQ) via a stepwise *in situ* growth strategy for tumor therapy through PTT and PDT (**Figure 8C**) 119. BQ was encapsulated into MIL-101, followed by the loading of catalase in the outer layer of MIL-101, to obtain octahedral spindle BQ-MIL@cat-MIL heterostructure with a particle size of ~140 nm. Subsequently, Cy 3-labeled caspase substrate peptide (Cy3-pep) and FA-modified PEG were anchored on the surface of BQ-MIL@cat-MIL to achieve active targeting and FL imaging capabilities. After entering tumor cells, the as-prepared BQ-MIL@cat-fMIL converted H_2_O_2_ into O_2_ via its outer layer, and then the produced O_2_ was transferred into the inner part to generate ^1^O_2_, which was beneficial to relieve tumor hypoxia and improve PDT effect. Synergistic PTT and PDT therapy results indicated that BQ-MIL@cat-fMIL exhibited a preeminent tumor therapeutic effect with the assistance of FL imaging.

Recently, Yang, Zhu, and coworkers synthesized a multifunctional nanoplatform, ICG-PtMGs@HGd, for multimodal imaging-guided tumor treatment by PTT and PDT (**Figure 8D**) 120. Pt NPs as nanoenzyme was dropped in the octahedral MOF scaffold, followed by the coating of porous Au nanoshell through a one-step reduction method. After anchoring the human serum albumin-chelated gadolinium (HGd) onto the outer surface, commercial NIR organic dye ICG was introduced in the pores of MOF to obtain ICG-PtMGs@HGd with good biocompatibility and tumor targeting effect. Experimental results proved that ICG-PtMGs@HGd had the abilities of O_2_ generation, passive/active tumor targeting, synergistic PTT and PDT, and multimodal imaging feature.

### 3.2 Multimodal therapy based on MOFs

#### Chemo/CDT/PTT

**Figure 9:**
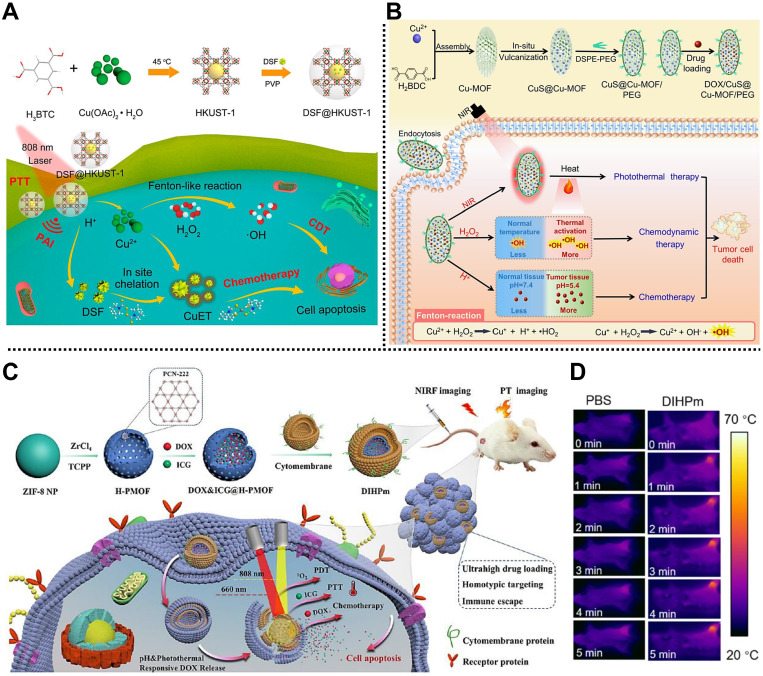
** (A)** Schematic diagram of the main synthetic route of DSF@HKUST-1 and the application for PAI -guided chemo/CDT/PTT of the tumor. Adapted with permission from 121. Copyright 2021, American Chemical Society. **(B)** The fabrication of DOX@CuS@Cu-MOF/PEG and its usage for synergistic chemo/CDT/PTT. Adapted with permission from 39. Copyright 2022, Elsevier. Schematic representation of **(C)** the preparation of hollow DIHPm NPs for imaging-guided synergistic chemo/PTT/PDT of the tumor, and **(D)** *in vivo* photothermal images of different treatment groups under NIR laser irradiation. Adapted with permission from 123. Copyright 2021, American Chemical Society.

Yang, Lin, and coworkers fabricated a simple nanoplatform based on Cu-MOF for PAI and tumor therapy through chemo/CDT/PTT (**Figure 9A**) 121. HKUST-1, composed of Cu^2+^ nodes and BTC organic ligands, was designed as a multifunctional nanocarrier to adsorb FDA-approved antitumor drug disulfiram (DSF), followed by the functionalization of PVP polymer to establish a spherical TME-responsive nanoplatform DSF@HKUST-1 with a DSF-loading amount of 12.60% and a photothermal conversion efficiency of 26.69%. Under the TME, DSF@HKUST-1 could be decomposed to release Cu^2+^ and DSF, triggering Cu^2+^-mediated CDT via the Fenton reaction and the DSF-induced chemotherapy. Similarly, another smart MOF-based nanocomposite constructed from CuS NPs, Cu-MOF, and PEG shell, namely DOX@CuS@Cu-MOF/PEG, was reported by Lian, Chen, and coworkers to load anticancer DOX for synergistic chemo/CDT/PTT (**Figure 9B**) 39. According to both *in vitro* and *in vivo* studies, tumor growth could be effectively inhibited by DOX@CuS@Cu-MOF/PEG through chemo/CDT/PTT with good biocompatibility, which enriches the toolbox of constructing simple MOF-based nanocomposites for multimodal tumor therapy.

#### Chemo/CDT/starvation therapy

For instance, Li and coworkers reported an intelligent nanocomposite with phosphate-responsibility, i.e., Mil-101(Fe)@GOx/DOX@TPP/FA (MGDFT NPs), for synergistic chemo/CDT/starvation therapy in 4T1 tumor-bearing mice 122. MIL-101(Fe) was used to decorate chemotherapeutic agent DOX and GOx and then coated with tumor target molecule GA-PEG and mitochondria target molecule (3-carboxypropyl)triphenylphosphonium bromide to construct polyhedral MGDFT NPs with an average size of ~150 nm and good targeting capability. Once the MGDFT NPs reached the tumor cell, they were degraded by the high concentrations of phosphate. Then, the released GOx could catalyze glucose to H_2_O_2_, triggering tumor starvation. Moreover, Fe^3+^ produced by MGDFT NPs degradation could convert H_2_O_2_ into •OH to enhance CDT efficiency. Furthermore, good tumor targeting capability allowed MGDFT NPs to be effectively internalized into tumor cells, showing enhanced therapeutic effects via chemo/CDT/starvation therapy with minor systemic toxicity.

#### Chemo/PTT/PDT

In collaboration with Tian and coworkers, we recently designed a biomimetic hollow MOF-based nanoplatform for imaging-guided synergistic chemo/PTT/PDT (**Figure 9C**) 123. Using ZIF-8 as a template, PCN-222 consisting of Zr^4+^ nodes and TCPP organic ligands was coated on the surface of ZIF-8 to form a core-shell MOF, and then the ZIF-8 template was removed by a self-sacrificial template method to obtain hollow MOF skeleton. After the encapsulation of anticancer drug DOX and photosensitizer ICG, the cancer cell membrane was coated on the outer surface to develop DIHPm NPs with outstanding homologous tumor-targeting, high loading, and immune escape capacities. Due to the ICG loading, the as-fabricated DIHPm NPs exhibited strong photothermal conversion ability and PDT effect under laser irradiation (**Figure 9D**). Meanwhile, DOX endowed DIHPm NPs with good chemotherapy ability. After intravenous administration into the 4T1 tumor-bearing mice, hollow DIHPm NPs showed good biocompatibility, homologous targeting, pH/photothermal responsive DOX release, and synergistic chemo/PTT/PDT effect under the guidance of photothermal and FL imaging.

**Figure 10:**
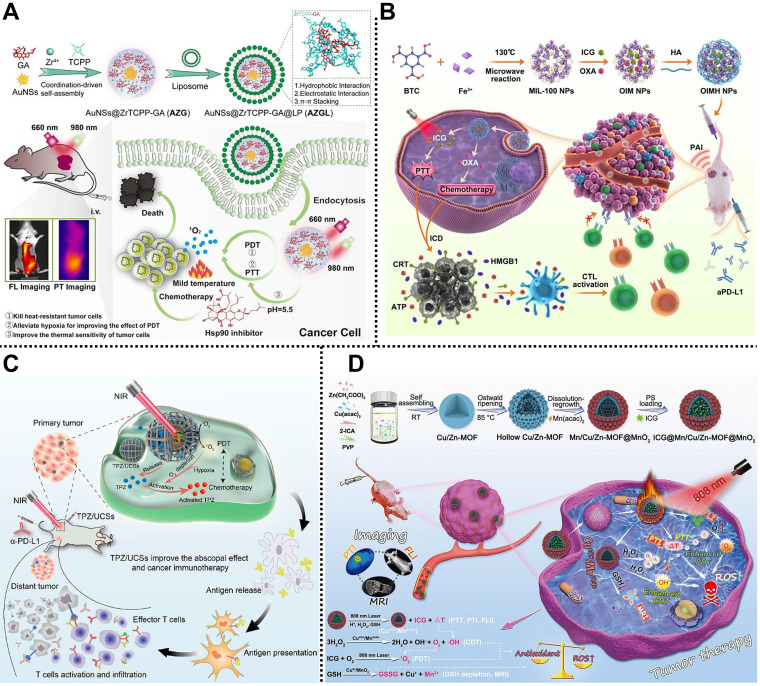
** (A)** Schematic diagram of the preparation of core-shell AZGL NPs and the therapeutic mechanism of the synergistic chemo/PTT/PDT. Adapted with permission from 124. Copyright 2022, Springer Nature. **(B)** Diagram of the design of OIMH NPs for application in the treatment of colon cancer by chemo/PTT/immunotherapy. Adapted with permission from 125. Copyright 2022, Elsevier. **(C)** The structure of TPZ/UCSs and the mechanism for chemo/PDT/immunotherapy. Adapted with permission from 126. Copyright 2020, American Chemical Society. **(D)** The fabrication of ICG@Mn/Cu/Zn-MOF@MnO_2_ for multimodal imaging-guided CDT/PTT/PDT. Adapted with permission from 127. Copyright 2021, Wiley-VCH Verlag GmbH&Co. KGaA, Weinheim.

Subsequently, Chen and coworkers reported a smart core-shell nanocomposite based on MOF and Au nanostar, AuNS@ZrTCPP-GA@LP (AZGL), for breast cancer therapy by chemo/PTT/PDT (**Figure 10A**) 124. In this system, heat shock protein 90 (HSP90) inhibitor of gambogic acid (GA) and photothermal agent Au nanostar were dropped in the MOF reservoir through a facile one-pot strategy, followed by the coating of PEGylated liposome to form AZGL bearing good biocompatibility and encapsulation rate of ~38.4%. According to the experimental results, AZGL demonstrated pH-dependent drug release, good tumor accumulation, and FL/photothermal imaging-guided combined chemo/PTT/PDT in the tumor.

#### Chemo/PTT/immunotherapy

Wang, Tian, Xu, and coworkers designed a multifunctional MOF-based nanoplatform for tumor treatment through chemo/PTT/immunotherapy (**Figure 10B**) 125. MIL-100 was synthesized via a microwave reaction to load NIR dye ICG and anticancer drug OxPt with high loading efficiency. Subsequently, HA was modified on the outer surface of MIL-100 to obtain OIMH NPs with a long-circulating capacity. The ICG and OxPt endowed the OIMH NPs with PTT and chemotherapy abilities, respectively. Meanwhile, OIMH NPs exhibited a sensitive PAI effect, which could be used for imaging-guided tumor therapy. Importantly, the OxPt and ICG in the OIMH NPs could stimulate immunogenic cell death, increase immune cell infiltration, and improve the number of effector memory T cells in the spleen, which was beneficial for recurrence suppression and precise treatment of tumors. Besides, this nanoplatform also showed the ability to induce potent antitumor immunity through immune escape suppression and immune activation in the CT26 bilateral tumor-bearing mice model, providing an alternative option for the efficient treatment of colon cancer.

#### Chemo/PDT/Immunotherapy

Li and coworkers synthesized an intelligent core-shell nanocomposite based on porphyrinic MOFs and UCNPs for combined treatment of hypoxic tumors through chemo/PDT/immunotherapy (**Figure 10C**) 126. In this design, porphyrinic PCN-224 as the shell was used for encapsulating the UCNPs core and the hypoxia-activated prodrug TPZ to construct TPZ/UCSs nanocomposite. Under NIR laser irradiation, the UCNP core in TPZ/UCSs could transfer NIR light energy to the porphyrin molecules in the MOF shell to generate ^1^O_2_, realizing efficient laser-triggered PDT of tumors. Moreover, the as-prepared TPZ/UCSs exhibited an enhanced chemotherapy effect at the tumor sites due to the hypoxia-activated feature of TPZ. Furthermore, combining chemo/PDT and immune checkpoint-blockade therapy could effectively prevent systemic tumor growth by generating specific cell-infiltrating cytotoxic T cells in CT26 tumor-bearing mice model. This work offers an option for efficient treatment of systemic tumors using multifunctional MOFs.

#### CDT/PTT/PDT

Yin and coworkers designed and fabricated a mixed porous Cu/Zn-MOF nanocomposite, ICG@Mn/Cu/Zn-MOF@MnO_2_, for multimodal imaging-guided CDT/PTT/PDT in U87 tumor-bearing mice (**Figure 10D**) 127. In this system, Cu^2+^, Zn^2+^, and imidazol-2-carboxyaldehyde (2-ICA) were used to prepare the mixed Cu/Zn MOF via self-assembly at room temperature. After the Ostwald ripening, the MnO_2_ shell was coated on the surface of Cu/Zn MOF to obtain ICG@Mn/Cu/Zn-MOF@MnO_2_ with the capability for loading ICG. Under NIR laser irradiation, the released ICG from the nanocomposite exhibited FL/photothermal imaging and PTT effect at the TME. Meanwhile, highly concentrated H_2_O_2_ could be catalyzed into O_2_ and toxic •OH through the Fenton reaction to improve CDT and PDT effect. Furthermore, the MnO_2_ shell on the surface of the nanocomposite could deplete GSH and generate Mn^2+^ to achieve MRI in the tumor sites. Notably, the mixed Cu/Zn-MOF possessed high ICG loading, low side effects, and enhanced synergistic CDT/PTT/PDT under the guidance of FL/MRI/photothermal imaging, paving the way for multimodal cancer theranostics assisted by MOF-based nanocomposites.

#### PTT/PDT/Immunotherapy

Recently, Liao, Qiao, Zhang, and coworkers reported a multimodal imaging-guided MOF-based nanoplatform for efficient treatment of tumor via PTT/PDT/immunotherapy in 4T1 tumor-bearing mice 128. Octahedral MIL-101-NH_2_ as the core carrier was used for grafted photoacoustic/fluorescent signal donor ICG through covalent bonding and load immune adjuvant CpG to develop multifunctional ICG-CpG@MOF bearing a diameter of approximately 150 nm and the loading efficiency of ~76% for CpG. The as-fabricated ICG-CpG@MOF could passively target tumor sites through EPR effects due to its suitable diameters. Moreover, CpG and the released tumor-associated antigen could induce the transformation of tumor cells from cold to hot by activating the immune system, improving the efficacy of tumor therapy. Experimental results demonstrated that biocompatible ICG-CpG@MOF not only exhibited multimodal imaging features including FL, PA, and MRI but also achieved efficient treatment of tumor PTT/PDT/immunotherapy under 808 nm laser irradiation, which provides a promising system for cancer theranostics.

## 4. Conclusion and outlook

In conclusion, we have summarized the recent advances in MOF-based nanocomposite as smart treatment nanoplatforms for efficient tumor therapy through single therapy, including RT, chemotherapy, CDT, phototherapy (PTT and PDT), starvation therapy, immunotherapy, and combination therapy methods. As aforementioned, the employment of multifunctional MOF-based nanocomposites as intelligent nanoplatforms exhibits distinct superiorities in cancer therapy compared with traditional cancer therapy modalities. As an emerging and fantastic class of porous nanomaterials, MOFs nanoplatforms have been widely exploited to deliver various functional agents, such as chemotherapeutic drugs, ions, phototherapeutic agents, proteins, enzymes, and antigens, for improving the therapeutic effect of cancer due to their diverse compositions and structures, tailorable morphologies, large surface areas and porosity, tunable pore size, easy functionalization, and good biocompatibility. Meanwhile, the passive and active targeting effects of MOF-based nanoplatforms provide a reliable guarantee for the efficient accumulation of therapeutic agents at tumor sites. To date, various smart MOF-based nanoplatforms have been designed and constructed to meet the urgent requirements of cancer therapy. Notably, among therapeutic strategies summarized in this review, MOF-based nanoplatforms also exhibit unique merits in sonodynamic therapy, microwave thermal therapy and dynamic therapy, gene therapy, and gas therapy, which have attracted extensive attention in recent decades. Many outstanding MOF-based nanoplatforms used in sonodynamic therapy 130, microwave thermal therapy and dynamic therapy 131, gene therapy 132, and gas therapy 133,134 have been designed and reported. Moreover, combination therapy employing multiple therapy strategies will overcome the drawbacks of single therapy to achieve precise treatment of tumors.

Although significant progress has been obtained in laboratory studies, MOF-based nanoplatforms are still confronted with significant challenges in the future development of clinical research in cancer therapy. Firstly, the fabrication of multifunctional MOF-based nanoplatforms usually involves complex synthesis and cumbersome steps during functionalization processes, which limit their clinical application to a great extent and require further validation of their significance in clinical transformation. Indeed, it is of great importance to construct MOF-based nanoplatforms with simple compositions and comprehensive functions for cancer therapy. Secondly, the toxicity and biosafety of MOF-based nanoplatforms are pivotal issues to be carefully emphasized in cancer therapy. Although numerous excellent studies have determined the toxicity of MOF-based nanoplatforms at the cellular and animal levels, most of them focus on short-term toxicity and ignore the long-term and acute toxicity of MOFs, which is adverse to broadening the application of MOFs in cancer therapy. Therefore, to comprehensively assess the toxicity of MOFs, long-term and acute toxicity experiments *in vivo* need to be performed 135. Notably, using ions with low toxicity and endogenous molecules as ligands to prepare MOFs is a feasible strategy to improve biocompatibility 32. Thirdly, the stability, aggregation, and premature clearance of MOF-based nanoplatforms during circulation are also crucial issues. The stability of MOFs in water and simulated physiological environments has been extensively conducted, but the complexity of natural physiological environments should be considered 77. Besides, severe aggregation of MOF-based nanoplatforms during circulation may trigger additional side effects and reduce cancer treatment efficacy. Moreover, premature clearance will reduce the accumulation of MOF-based nanoplatforms at target sites, thus inducing the therapeutic effect. Generally, size control and surface functionalization are used to conquer these shortcomings. Fourthly, the efficient anticancer effect of MOFs has been demonstrated in animal models, such as mice and rabbits, but their effects on big animals and humans remain to be further investigated. Finally, the metabolic mechanism and degradation pathway of MOF-based nanoplatforms need to be profoundly and systematically studied. Although many researchers have monitored the degradation process of MOFs during a single treatment by combining imaging strategies, comprehensively understanding the clearance mechanism of MOFs is still very difficult. Multiple administrations are often required for tumor therapy, indicating that the MOFs clearance mechanisms and degradation pathways require long-term monitoring to obtain comprehensive theoretical support.

Overall, although MOF-based nanoplatforms still face various challenges in cancer therapy, the significant progress achieved so far has facilitated their further applications. Given the rapid development of multidisciplinary technologies in recent decades, multifunctional MOF-based nanoplatforms will be further developed in cancer therapy by employing therapy methods from single to combination therapy methods, thus boosting the anticancer effect and endowing the patients with improved quality of life.

## Figures and Tables

**Table 1:** Examples of MOF-based nanoplatforms used in single cancer therapy including RT, chemotherapy, CDT, phototherapy, starvation therapy, and immunotherapy

MOF	Compositions	Therapeutic method	*In vivo* model	Administration type	Ref.
Zr-MOF-QU	Zr^4+^, BDC	RT	A549 tumor-bearing BALB/c nude mice	Intravenous injection	41
MnTCPP-Hf-FA	Hf^4+^, TCPP	RT	B16-F10 tumor-bearing mice	Intravenous injection	42
UAAP	Zr^4+^, PTA	RT	MDA-MB-231 tumor-bearing mice	Intravenous injection	38
UiO-66-NH_2_(Hf)	Hf^4+^, NH_2_-BDC	RT	KYSE 150 tumor-bearing nude mice	Intratumoral injection	43
CP5-capped UMCM-1-NH-Py	Zn^2+^, NH_2_-BDC, BTB	Chemotherapy	—	—	44
CP5-capped UiO-66-NH-Q	Zr_6_, NH_2_-BDC	Chemotherapy	—	—	45
CP5-capped UiO-66-NH-A	Zr_6_, NH_2_-BDC	Chemotherapy	—	—	46
TYR@NPCN-333(Al)	Al^3+^, TATB	Chemotherapy	HeLa tumor-bearing mice	Intratumoral injection	47
DOX@MOFs-Glu	Gd/Yb, BBDC	Chemotherapy	HeLa tumor-bearing mice	Intravenous injection	48
ZIF-8-DOX-LY-RM	Zn^2+^, 2-MIM	Chemotherapy	4T1 tumor-bearing mice	Intravenous injection	49
ClO@MOF/68	Zn^2+^, 2-MIM	CDT	4T1 tumor-bearing mice	Intravenous injection	50
Co-Fc@GOx	Co^2+^, Fc(COOH)_2_	CDT	4T1 tumor-bearing mice	Intratumoral injection	51
PZIF-67-AT	Co^2+^, 2-MIM	CDT	H22 tumor-bearing mice	Intravenous injection	52
DHA@MIL-101	Fe^3+^, NH_2_-BDC	CDT	Lewis lung cancer-bearing mice	Intravenous injection	53
DBP-UiO	Hf^4+^, H_2_DBP	PDT	SQ20B tumor-bearing mice	Intratumoral injection	54
DBC-UiO	Hf^4+^, H_2_DBC	PDT	CT26 and HT29 tumor-bearing mice	Intratumoral injection	55
FA-PCN-224	Zr_6_, H_2_TCPP	PDT	—	—	56
aMMTm	Zr^4+^, H_2_TCPP	PDT	4T1 tumor-bearing mice	Intravenous injection	57
OxgeMCC-r SAE	Mn^2+^, [Co(C≡N)_6_]	PDT	4T1 tumor-bearing mice	Intravenous injection	58
UMOFs@Au	Zr^4+^, TCPP-Fe	PDT	U87MG tumor-bearing mice	Intravenous injection	59
MOF@HA@ICG	Fe^3+^, BTC	PTT	MCF-7 tumor bearing mice	Intravenous injection	60
UiO-66@CyP	Zr^4+^, BDC	PTT	CT26 tumor-bearing mice	Intratumoral injection	61
Cy@ZIF-8	Zn^2+^, 2-MIM	PTT	U14 tumor-bearing mice	Intratumoral injection	62
AuNS@MOF-ZD2	Fe^3+^, NH_2_-BDC	PTT	MDA-MB-231 tumor-bearing mice	Intravenous injection	63
Gd-DTPA-MOF-808@PANI	Zr_6_, BTC	PTT	4T1 tumor-bearing mice	Intravenous injection	64
TGZ@eM	Zn^2+^, 2-MIM	Starvation therapy	CT26 tumor-bearing mice	Intravenous injection	65
HZ@GD	Zn^2+^, 2-MIM	Starvation therapy	B16-F10 tumor-bearing C57BL/6 mice	Intravenous injection	66
OVA@ZIF-8-CpG	Zn^2+^, 2-MIM	Immunotherapy	Kunming mice	Subcutaneous injection	67
MOF-OVA@CpG	Eu^3+^, GMP	Immunotherapy	B16-F10 tumor bearing mice	Subcutaneous injection	68
MOF-gated MS	Zn^2+^, 2-MIM	Immunotherapy	E.G7-OVA tumor-bearing mice	Subcutaneous injection	69
LYS-NPs	Zn^2+^, 2-MIM	Immunotherapy	4T1 tumor-bearing mice	Intravenous injection	70

**Table 2:** Summary of the features and mechanisms of the methods used for cancer therapy

Therapeutic strategies	Therapeutic principle	Superiorities	Drawback
RT	Ray irradiation	Deep penetration, local treatment, and good elimination capability	Serious side effects, and easy to cause radiation resistance
Chemotherapy	Chemotherapeutic agents	Wide application range and good therapeutic effect	Low bioavailability, serious side effects, and easy to generate multidrug resistance
CDT	Fenton and Fenton-like reaction	O_2_, external energy, and device independence, high specificity, and low toxicity	Highly dependent on H_2_O_2_ concentration
PTT	Turns NIR laser into hyperthermia	Non-invasiveness, high controllability, O_2_ independence, and negligible side effects	Low specificity and limited penetration of light
PDT	Light-induced ROS/^1^O_2_ generation	Low toxic and side effects, non-invasiveness, and high selectivity	Limited light depth, high dependence on O_2_ and specific device
Starvation therapy	Consumption of nutrients	Non-invasiveness, low side effects, and device independence	Hypoxia aggravation in the TME, high dependence on O_2_
Immunotherapy	Induces immune response	Wide application range, low side effects, and triggerable memory effect	Inflammatory response, complex immunosuppression TME, and atypical clinical reaction rates

**Table 3:** Examples of MOF-based nanoplatforms for combination cancer therapy, including bimodal and multimodal therapy

MOF	Composition	Therapeutic methods	*In vivo* model	Administration type	Ref.
PEG-Au/FeMOF@CPT	Zr^4+^, TCPP-Fe	Chemotherapy/CDT	HepG2 tumor-bearing mice	Intravenous injection	110
PUWPFa	Zr^4+^, NH_2_-BDC	Chemotherapy/PTT	HeLa tumor-bearing mice	Intravenous injection	40
AuMC	Fe^3+^, NH_2_-BDC	Chemotherapy/PTT	A2780 tumor-bearing mice	Intravenous injection	111
Au@MOF-DOX	Zn^2+^, 2-MIM	Chemotherapy/PTT	H22 tumor bearing mice	Intravenous injection	112
ALA@UiO-66NH-FAM@CP1	Zr^4+^, NH_2_-BDC	Chemotherapy/PDT	HeLa tumor-bearing mice	Intravenous injection	113
DGZ	Zn^2+^, 2-MIM	Chemo/starvation therapy	4T1 tumor-bearing mice	Intravenous injection	114
RPMANB	Zr^4+^, BDC, BCN3	Chemo/immunotherapy	4T1 tumor-bearing mice	Intravenous injection	115
Dox@MOF-Au-PEG	Zr^4+^, H_2_TCPP	Chemoradiotherapy	U87MG tumor-bearing mice	Intravenous injection	116
RCDs@MIL-100	Fe^3+^, BTC	CDT/PTT	4T1 tumor-bearing mice	Intravenous injection	117
mCGP	Zr_6_, H_2_TCPP	PDT/starvation therapy	4T1 tumor-bearing mice	Intravenous injection	118
BQ-MIL@cat-fMIL	Fe^3+^, NH_2_-BDC	PTT/PDT	HeLa tumor-bearing mice	Intravenous injection	119
ICG-PtMGs@HGd	Zr^4+^, NH_2_-BDC	PTT/PDT	4T1 tumor-bearing mice	Intravenous injection	120
DSF@HKUST-1	Cu^2+^, BTC	Chemo/CDT/PTT	4T1 tumor-bearing mice	Intravenous injection	121
DOX@CuS@Cu-MOF/PEG	Cu^2+^, BDC	Chemo/CDT/PTT	4T1 tumor-bearing mice	Intratumoral injection	39
MGDFT NPs	Fe^3+^, BDC/NH_2_-BDC	Chemo/CDT/starvation therapy	4T1 tumor-bearing mice	Intravenous injection	122
DIHPm	Zr^4+^, TCPP	Chemo/PTT/PDT	4T1 tumor-bearing mice	Intravenous injection	123
AZGL	Zr^4+^, TCPP	Chemo/PTT/PDT	4T1 tumor-bearing mice	Intravenous injection	124
OIMH	Fe^3+^, BTC	Chemo/PTT/immunotherapy	CT26 tumor-bearing mice	Intravenous injection	125
TPZ/UCSs	Zr_6_, H_2_TCPP	Chemo/PDT/immunotherapy	CT26 tumor-bearing mice	Intravenous injection	126
ICG@Mn/Cu/Zn-MOF@MnO_2_	Cu^2+^, Zn^2+^, 2-ICA	CDT/PTT/PDT	U87 tumor-bearing mice	Intravenous injection	127
ICG-CpG@MOF	Fe^3+^, NH_2_-BDC	PTT/PDT/immunotherapy	4T1 tumor-bearing mice	Intravenous injection	128

## Abbreviations

MOFs: metal-organic frameworks
RT: radiotherapy
CDT: chemodynamic therapy
TME: tumor microenvironment
PDT: photodynamic therapy
PTT: photothermal therapy
EPR: enhanced permeability and retention
NPs: nanoparticles
BDC: 1,4-benzenedicarboxylic acid
QU: quercetin
BSA: bovine serum albumin
CA IX: carbonic anhydrase IX
TCPP: tetrakis(4-carboxyphenyl) porphyrin
FA: folic acid
ROS: reactive oxygen species
PTA: *p*-phthalic acid
ASO: antisense oligonucleotide
PEG: polyethylene glycol
NH_2_-BDC: 2-amino-terephthalic acid
PBS: phosphate-buffered saline
DMEM: Dulbecco's modified Eagle's medium
DOX: doxorubicin
5-Fu: 5-fluorouracil
OxPt: oxaliplatin
APAP: paracetamol
UMCM: University of Michigan Crystalline Material
CP5: carboxylatopillar[5]arene
BTB: 4,4',4''-benzene-1,3,5-triyl-tribenzoic acid
Q: quaternary ammonium
TYR: tyrosinase
TATB: 4,4',4''-s-triazine-2,4,6-triyl-tribenzoic acid
GSH: glutathione
BBDC: 5-boronobenzene-1,3-dicarboxylic acid
2-MIM: 2-methylimidazole
F68: poloxamer 188
FC: Co-ferrocene
3-AT: 3-amino-1,2,4-triazole
DHA: monomer-dihydroartemisinin
H_2_DBC: 5,15-di(*p*-benzoato)-chlorin
SEM: scanning electron microscopy
H_2_TCPP: tetrakis(4-carboxyphenyl)porphyrin
VEGFR2: vascular endothelial growth factor receptor 2
MRI: magnetic resonance imaging
UCNPs: upconversion nanoparticles
TCPP-Fe: Fe(III) *meso*-tetrakis(4-carboxyphenyl)porphine chloride
NIR: near-infrared
HA: hyaluronic acid
BTC: 1,3,5-benzenetricaboxylic acid
ICG: indocyanine green
FL: fluorescence
PAI: photoacoustic imaging
CyP: cyanine-containing polymer
Cy: cyanine
PVP: polyvinylpyrrolidone
PANI: polyaniline
GOx: glucose oxidase
TPZ: tirapazamine
OVA: ovalbumin
IL-1: interleukin-1
IL-6: interleukin-6
IFN-γ: interferon-γ
TNF-α: tumor necrosis factor-α
TEM: transmission electron microscope
GMP: guanine monophosphate
MS: mesoporous silica
CPT: camptothecin
AuNRs: gold nanorods
CT: computed tomography
5-FAM: 5-carboxyl fluorescein
5-ALA: 5-aminolevulinic acid
BCN3: 4-azidobenzoic acid
BQ: black phosphorus quantum dot
DSF: disulfiram
HSP90: heat shock protein 90
GA: gambogic acid
RCDs: NIR emission carbon dots
2-ICA: imidazol-2-carboxyaldehyde
